# Radiomics for Identification and Prediction in Metastatic Prostate Cancer: A Review of Studies

**DOI:** 10.3389/fonc.2021.771787

**Published:** 2021-11-01

**Authors:** Jake Kendrick, Roslyn Francis, Ghulam Mubashar Hassan, Pejman Rowshanfarzad, Robert Jeraj, Collin Kasisi, Branimir Rusanov, Martin Ebert

**Affiliations:** ^1^ School of Physics, Mathematics and Computing, University of Western Australia, Perth, WA, Australia; ^2^ Medical School, University of Western Australia, Crawley, WA, Australia; ^3^ Department of Nuclear Medicine, Sir Charles Gairdner Hospital, Perth, WA, Australia; ^4^ Department of Medical Physics, University of Wisconsin, Madison, WI, United States; ^5^ Faculty of Mathematics and Physics, University of Ljubljana, Ljubljana, Slovenia; ^6^ Department of Radiation Oncology, Sir Charles Gairdner Hospital, Perth, WA, Australia; ^7^ 5D Clinics, Claremont, WA, Australia

**Keywords:** radiomics, metastatic prostate cancer, radiomics quality score, deep learning, PET, CT, MRI

## Abstract

Metastatic Prostate Cancer (mPCa) is associated with a poor patient prognosis. mPCa spreads throughout the body, often to bones, with spatial and temporal variations that make the clinical management of the disease difficult. The evolution of the disease leads to spatial heterogeneity that is extremely difficult to characterise with solid biopsies. Imaging provides the opportunity to quantify disease spread. Advanced image analytics methods, including radiomics, offer the opportunity to characterise heterogeneity beyond what can be achieved with simple assessment. Radiomics analysis has the potential to yield useful quantitative imaging biomarkers that can improve the early detection of mPCa, predict disease progression, assess response, and potentially inform the choice of treatment procedures. Traditional radiomics analysis involves modelling with hand-crafted features designed using significant domain knowledge. On the other hand, artificial intelligence techniques such as deep learning can facilitate end-to-end automated feature extraction and model generation with minimal human intervention. Radiomics models have the potential to become vital pieces in the oncology workflow, however, the current limitations of the field, such as limited reproducibility, are impeding their translation into clinical practice. This review provides an overview of the radiomics methodology, detailing critical aspects affecting the reproducibility of features, and providing examples of how artificial intelligence techniques can be incorporated into the workflow. The current landscape of publications utilising radiomics methods in the assessment and treatment of mPCa are surveyed and reviewed. Associated studies have incorporated information from multiple imaging modalities, including bone scintigraphy, CT, PET with varying tracers, multiparametric MRI together with clinical covariates, spanning the prediction of progression through to overall survival in varying cohorts. The methodological quality of each study is quantified using the radiomics quality score. Multiple deficits were identified, with the lack of prospective design and external validation highlighted as major impediments to clinical translation. These results inform some recommendations for future directions of the field.

## 1 Introduction

Prostate Cancer (PCa) is a pernicious disease that is one of the leading causes of cancer death among men throughout the world (1). Early-stage diagnosis yields high 5-year survival rates of above 90%, however, once the disease metastasises, it becomes very lethal as 5-year survival rates drop drastically to less than 40% (2). For patients with bone metastases (BM), which is one of the most common sites of metastases in PCa (3), 5-year survival rates drop even further to less than 10% (4). Lymph node involvement (LNI) also yields a poor prognosis for patients (5). The clinical management of the disease is complicated by its heterogeneity (6), with current practice aiming to stratify patients into risk categories such that high-risk lesions are identified and treated, and the over-treatment of low-risk lesions is minimized (7). Radical prostatectomy (RP) is a prominent treatment for localised disease, however, between 20 and 40% of patients presents with biochemical recurrence (BCR) with the possibility of developing subsequent metastasis (8). The early identification of localised PCa patients at high risk of developing subsequent nodal or distant metastases is thus crucially important and can substantially affect the clinical decision-making process to the potential benefit of the patient (9). Established techniques for PCa diagnosis and risk stratification such as the digital rectal examination (DRE), prostate-specific antigen (PSA) test, and transrectal ultrasound (TRUS)-guided biopsy have significant limitations. DRE suffers from a high false positive rate (10), while PSA is a non-specific blood biomarker that can be elevated even in the absence of PCa (11), and even low levels do not preclude the presence of high- or medium-grade PCa (12). TRUS-guided biopsy is typically conducted *via* random sampling and fails to capture the heterogeneity inherent in the lesion. Furthermore, analysis of post RP specimens has demonstrated that the Gleason Score (GS) obtained from the pre-treatment needle biopsy often differs from that obtained on the final RP specimen (13).

The potential for non-invasive assessment of PCa risk, metastatic potential, and even treatment response, has been facilitated by the advance of medical imaging technologies in recent decades. Medical imaging modalities such as multiparametric magnetic resonance imaging (mpMRI), positron emission tomography (PET) and computed tomography (CT) play an important role in the diagnosis and management of localised and metastatic prostate cancer (mPCa). Bone scintigraphy is an important imaging modality that is commonly used to diagnose the extent of bone metastasis in mPCa patients (14). mpMRI is the most important imaging modality in the initial detection and staging of localised PCa due to its superior soft tissue contrast and high resolution and can localise areas of suspicion for subsequent biopsy (15, 16). PET radiotracers that target prostate specific membrane antigen (PSMA) such as ^68^Ga-PSMA are quickly becoming the standard of care when it comes to the management of biochemically recurrent PCa following definitive primary therapy. PSMA tracers enable detection of suspicious lesions because they target directly the PSMA receptor which is vastly overexpressed in the majority of PCa cases (17–19). The quantitative, rather than qualitative, analysis of each of these imaging modalities to identify unique diagnostic or prognostic biomarkers has the potential to become the basis for a personalised medicine approach to patient treatment.

Radiomics is defined as the extraction of large numbers of imaging features from medical images to quantify specific tumour attributes and phenotypes, with the ultimate goal of utilising these features to glean valuable diagnostic or prognostic information that can inform clinical decision making (20). The central hypothesis of radiomics is that these extracted mathematical features are reflective of the underlying tumour biology, and that they can therefore be used to guide treatment procedures and advance personalised therapy on a patient-to-patient basis (21). Typically, images are visually assessed by radiologists whose qualitative observations are highly variable (22–24). The extraction of radiomics features, combined with a sufficiently quantitative image acquisition, enables a more quantitative and objective characterisation of the tumour which can overcome this inter-observer variability, and potentially yield useful predictive biomarkers that cannot be discerned *via* visual analysis. The radiomics approach has the advantage of being non-invasive, as opposed to other techniques such as biopsy which, in addition to being invasive (25), are limited in their capacity to characterise the spatial and temporal heterogeneity of lesions (26). Non-invasive assessment of intra-tumoral heterogeneity is highly desirable since it is a known factor affecting disease progression and response (27). Furthermore, medical imaging scans are a part of the conventional clinical management scheme for most patients, meaning that radiomics models can typically be incorporated clinically without adding any significant burden to the existing workflow (28).

Despite the enormous potential of radiomics to facilitate individualised patient therapy, the process does come with associated challenges. The radiomics workflow contains a myriad of factors that can profoundly affect the resulting quantitative imaging biomarker measurement; anything from the algorithm used to reconstruct the medical image to the interpolation method utilised in an up- or down-sampling procedure can introduce variability into radiomics research (29, 30). The sensitivity of extracted features to a host of procedural factors has contributed to poor scientific reproducibility in radiomics research that substantially affects its translational capacity (31). Recognising the need to ensure the scientific rigour of radiomics studies, Lambin et al. (32) introduced the Radiomics Quality Score (RQS), a points-based system that rewards and penalises radiomics papers according to specific attributes of their methodologies. Modelled on the Transparent Reporting of a multi-variable prediction model for Individual Prognosis or Diagnosis (TRIPOD) initiative (33), the RQS identifies key aspects of the radiomics workflow, such as the necessity of reporting imaging parameters in a comprehensive fashion, conducting external validation of the results and the nature of the study (retrospective/prospective), and generates a score out of 36 providing an indication of how scientifically rigorous the study is. The RQS, despite some limitations, is a useful way to identify methodological weaknesses in reported radiomics studies. The quality of radiomics reporting, as measured by the RQS, in oncological studies in general is poor (34), and a plethora of studies have demonstrated that this fact generalises across a range of malignancies and modalities such as liver metastases (35), prostate cancer MRI studies (36), neuro-oncologic studies (37), and non-small cell lung cancer radiomics research (38). No such analysis has been undertaken for radiomics studies pertaining to mPCa.

This review aims to, (i) provide a methodological overview of the radiomics workflow with comments on how artificial intelligence (AI) techniques can complement the process, (ii) elucidate the current landscape of literature pertaining to how radiomics models can potentially be utilised in the clinical management of mPCa while providing a quality assessment in the form of a RQS and, (iii) comment on the limitations of the field and offer recommendations on future research.

## 2 Radiomics: A Methodological Overview

**Figure 1:**
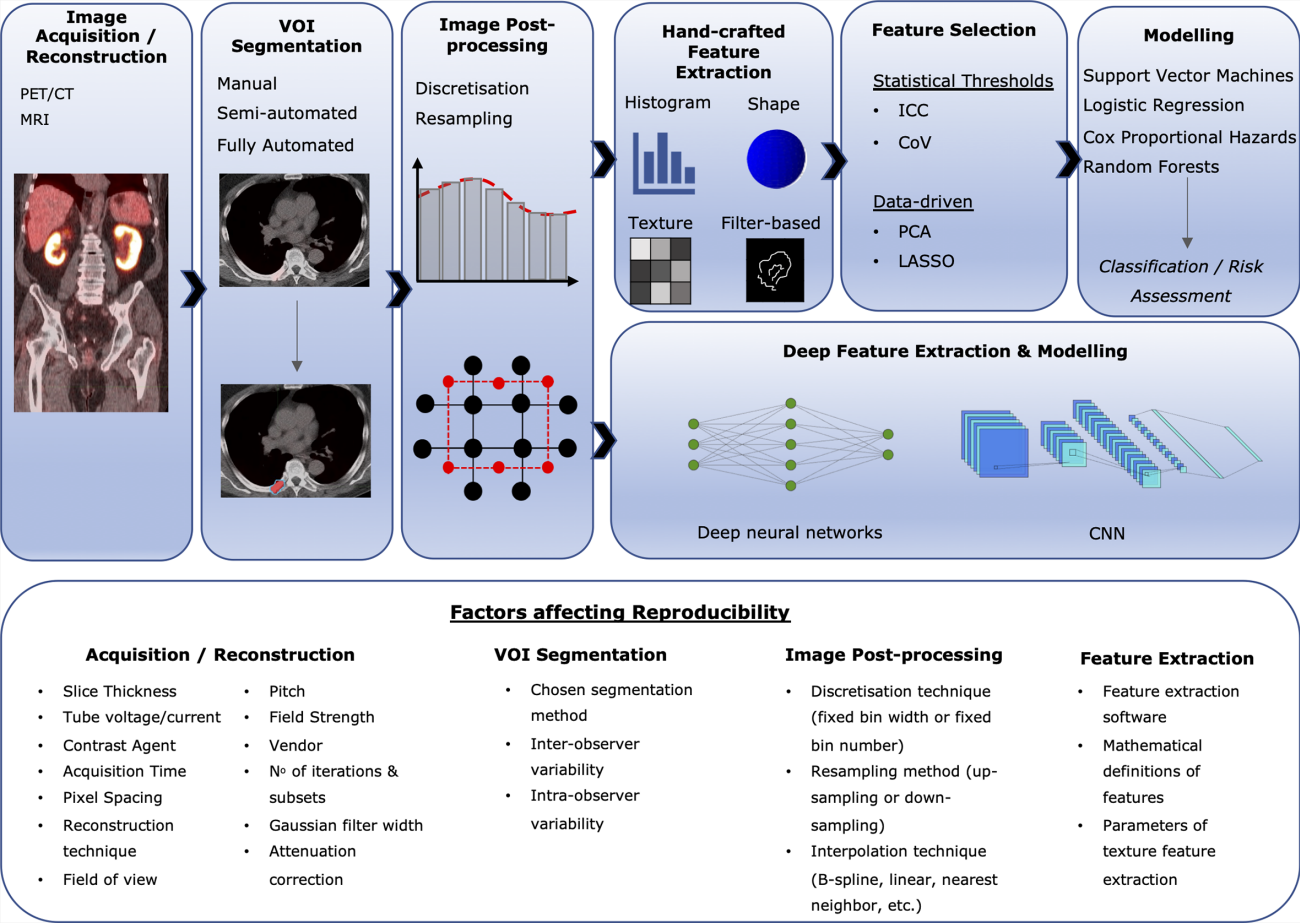
Overview of the radiomics workflow, with a non-exhaustive list of some critical factors affecting feature reproducibility.

The typical radiomics workflow consists of several defined steps, including: (i) Medical image acquisition and reconstruction; (ii) Region of Interest (ROI) segmentation; (iii) post-processing of the acquired image; (iv) feature extraction; (v) feature selection; and (vi) model development. These steps are summarised in **Figure 1**. Critical factors affecting the numerical output of each feature will be discussed where appropriate as each step is outlined sequentially below. The applicability of AI methods and how they can substantively aid the process will also be discussed.

### 2.1 Image Acquisition/Reconstruction

The first step in the radiomics pipeline is the acquisition of a medical image, which becomes the basis for the analysis conducted throughout the rest of the process. The acquisition and reconstruction of medical images is subject to significant variability both within and between different institutions. In theory, any parameter that will affect the output distribution of voxel intensities will affect the calculated feature values, and thus the medical image acquisition and reconstruction parameters will greatly affect the outcome of the feature extraction process. This remains true regardless of the medical imaging modality used on the patient and can have a significant effect on the reproducibility of radiomics studies: features that demonstrate clinical relevance in one clinical setting may not be useful in a separate institution where a different imaging protocol is used. The slice thickness and pixel spacing of reconstructed medical images, for example, are known factors affecting radiomics feature output values (39, 40). A recent systematic review identified the reconstruction algorithm, number of iterations, and the level of gaussian smoothing as factors also affecting biomarker reproducibility for PET scans (41). When acquiring CT scans of patients, acquisition variables such as the tube current and voltage (42, 43), the pitch (44), and even the vendor of the scanner can affect the numerical output of calculated features (45, 46). MRI is particularly challenging as a modality since voxel intensity values are not standardised and can vary greatly depending on the acquisition parameters chosen (47), and studies have indeed demonstrated that some of these parameters, such as image noise (48), choice of reconstruction algorithm (49), dynamic range and matrix size (50), do affect the output feature values. **Figure 1** displays a non-exhaustive list of the critical factors to consider that will affect biomarker outputs at each step in the radiomics workflow. The large dependence of radiomics features on acquisition and reconstruction protocols makes it imperative that these protocols are extensively documented when presenting the results of radiomics research to maximise study reproducibility (32).

### 2.2 ROI Segmentation

A precise delineation of the ROI is a requirement for input into the feature extraction algorithm. The image voxels within this ROI define the anatomical/physiological area from which the features will be extracted in subsequent steps in the radiomics workflow; therefore, any variability in this segmentation will affect the numerical output for each feature. Segmentation of ROI’s can be done manually, semi-automatically or automatically. Manual segmentations performed by clinical experts are typically used, however, performing this task manually has well documented limitations such as inter-observer variability and a significant clinical time burden, which limits its feasibility for radiomics analyses in larger datasets (51, 52). Variability in manual segmentations has the effect of introducing bias into the evaluation of quantitative imaging biomarkers (53). Efforts should be taken to mitigate against this bias by performing multiple segmentations. Feature robustness analyses should be conducted both between multiple independent manual observer segmentations, and also between manual segmentations and semi- or fully automated algorithms, where possible (32).

### 2.3 Image Post-Processing

Prior to radiomics feature analysis, there are a number of imaging post-processing steps that are typically conducted. Image discretization is one of these steps, involving the discretization of image voxel intensities from a continuous spectrum into a set of discrete intensity bins. Limiting the range of intensity values is necessary to make the calculation of subsequent radiomics features computationally tractable (22). It also has the benefit of noise reduction (54). There are two discretisation schemes available to radiomics researchers, namely, fixed bin number and fixed bin width, and the method chosen can affect the quantitative metrics subsequently extracted. A plethora of studies have demonstrated that the intensity discretisation scheme used can affect the reproducibility of radiomics features, and thus potentially affect the predictive model derived from them (40, 55–59). This fact, among many others discussed below, underscores the critical importance of transparent and comprehensive reporting of post-processing steps undertaken in radiomics studies.

Resampling image voxels to isotropic spacing is another necessary post-processing technique. Isotropic voxel spacing is necessary to ensure that the extracted texture features are rotationally invariant (54). Moreover, in-plane and through-plane spatial resolutions of medical images are commonly not unified across patient scans in radiomics datasets which can affect output feature values; therefore, resampling to a common spatial voxel resolution can be employed in an attempt to increase the reproducibility of feature values (30). There is as yet no consensus on whether up-sampling or down-sampling is preferred. Down-sampling images to a lower spatial resolution will necessarily result in information loss, whereas up-sampling images will result in the addition of false information. Different interpolation techniques exist to resample images to isotropic voxel spacing, such as nearest neighbour, trilinear, and cubic B-Spline; the method chosen can have a significant impact on the reproducibility of radiomic features. Recent studies have demonstrated that the chosen interpolation technique affects the number of reproducible features across all of the common modalities used in mPCa imaging, such as CT (60), PET (61), and MRI (62). Open and comprehensive reporting of the technique used is therefore necessary to ensure study reproducibility.

### 2.4 Feature Extraction

The crux of radiomics is the extraction of features from medical images. These features become the basis for which diagnostic and prognostic predictive models are generated which can be utilised to inform clinical decision making that is personalised to the specific biological attributes of the patient. In conventional radiomics practice, mathematically defined features that are hand-crafted using domain knowledge numbering in the hundreds, or sometimes thousands, are extracted from the ROI. However, with the recent surge in deep learning-based models and their applicability to medical images, it has become possible to mitigate the use of hand-crafted features and train complex neural network (NN) and convolutional neural network (CNN) models that are capable of learning the most salient features in unsupervised or supervised manners (63, 64). These two distinct types of features extracted from medical images will henceforth be referred to as *hand-crafted* features and *machine-learnt* features, respectively.

Hand-crafted features have the ability to capture either spatial or temporal heterogeneities within the defined ROI and can thus be categorised broadly as being either static or dynamic (65). Static radiomic features are time invariant and therefore characterise only spatial properties of the tumour. They are comprised of shape-based (morphological) and statistical features, which are further divided into first-, second-, and higher-order outputs. Morphological features describe the geometry of the lesion such as its compactness, sphericity, or surface to volume ratio (66, 67). First-order statistical features are derived from first-order histograms describing the distribution of voxel intensities within the specified tumour volume. Second-order statistical features, or what are often referred to as ‘texture features’, are among the most common descriptors used in radiomics predictive modelling. Texture features are able to characterise intensity spatial interrelationships between tumour voxels that first-order statistics fail to capture (65). Texture features can be extracted from a variety of defined matrices, such as the grey-level co-occurrence matrix (GLCM) (68), the grey-level run length matrix (GLRLM) (69), the grey-level size zone matrix (GLSZM) (70), the neighbourhood grey-tone difference matrix (NGTDM) (71), and the neighbourhood grey-level dependence matrix (NGLDM) (72), for use in predictive modelling. Higher-order statistical features involve the application of various mathematical transformations to the original image from which additional statistical features of first- and second-order can be extracted (21, 73), such as Laplacian of gaussian transformations, wavelet decompositions, and gabor filters for edge detection (74, 75). Dynamic features capture temporal information about the evolution of the disease over time that can provide more information than a simple snapshot of a lesion at a single time point. These features might unearth new biological characteristics of tumours that can be used in predictive modelling (76). There are, then, a very large array of possible features that can potentially be extracted from the ROI during a radiomics study. In practice, these features need not all be manually defined by the researcher, as the procedure of feature extraction can be performed by a number of commercial and open-source projects dedicated to the task.

Deep NN’s and CNN’s can be used to automatically learn high-level representations of input data such as medical images and generate machine learnt features. These techniques can be used to perform end-to-end predictive modelling tasks, encompassing automated hierarchical feature extraction and the utilisation of these features for the subsequent classification or regression task in a single step, or alternatively be used as standalone feature extractors (63). Deep CNN’s, for example, involve the repeated convolution of learnable filter grids across an input medical image whose values are tuned during the network training process to minimise a cost function such that the salient features relevant to the clinical task at hand are extracted. In this fashion, features are engineered automatically in a hierarchical way where simple characteristics are detected in the lower layers of the network, and increasingly abstract representations of the input image are learned as it progresses deeper through the network architecture (77, 78). There is a growing body of evidence demonstrating the usefulness of CNN-based features as a concomitant to traditional hand-crafted features in radiomics studies pertaining to various malignancies, such as soft tissue sarcoma (79), glioma (80), lung cancer (81), and also mPCa (82). Deep learning feature generators have greater versatility by not limiting themselves exclusively to manual human-defined features, however, this comes at the significant cost of reduced interpretability due to the black box nature of these algorithms. The benefits should therefore be weighed against the limitation of model interpretability, which is often desirable in the clinical context (83).

### 2.5 Feature Selection

The dimensions of the extracted feature space may be large relative to the patient sample size, partly due to the nature of medical research where ethical considerations constrain access to patient data. Generating a predictive model with more explanatory variables than patient samples prevents generalisability of the model by over-fitting the sample on which the model was trained (83, 84). Reducing the dimensionality of the feature space improves the prediction capabilities of the final model, increases model interpretability, and shortens the training time. Selecting a subset of good features is therefore a crucial part of the radiomics workflow. Feature selection can be conducted according to the calculation of traditional statistical measures where features are eliminated through the application of thresholds, or, the dimensions of the feature space can be reduced by data-driven algorithms that project the data into lower dimensional spaces. Through the conduction of the feature selection process, quantitative imaging biomarkers should be chosen based on the possession of the following properties: repeatability, non-redundancy and reproducibility. Reproducibility has been stressed in the sections above, the main considerations being feature reproducibility with respect to segmentation and scanner variability.

Where possible, available test-retest data should be utilised to assess the repeatability of features across imaging scans taken under identical conditions (85). It is common to select arbitrary thresholds based on, for example, intraclass correlation coefficient (ICC) metrics calculated between the test-retest biomarker outputs, to exclude non-repeatable features (58, 86). This simple thresholding method is widely used, but its drawbacks should be noted. Firstly, comparisons of ICC values between different populations are invalid since the metric depends on the variance of the data, and thus also the underlying characteristics of the population under analysis (87, 88). Secondly, repeatability analysis alone is insufficient to determine the usefulness of a quantitative imaging biomarker. In particular, when it comes to response assessment, a feature with low repeatability may change drastically in response to therapy and could therefore be more informative as a predictive biomarker than a feature with high repeatability that changes only minimally during the same treatment. Therefore, it is not appropriate in all cases to remove non-repeatable features based on cut-off values of repeatability metrics. This point has been argued by Lin et al. (89), who posit a new metric for longitudinal assessment of predictive biomarkers termed the ‘response-to-repeatability’ ratio which weighs the biomarker sensitivity (measured as a change between baseline and follow-up scan) against its repeatability.

Redundant features that are highly correlated with each other are unlikely to provide any additional information useful for predictive modelling and can lead to model instability (90). Furthermore, their inclusion can increase the chances of overfitting and hamper model generalisability. Clusters of highly correlated features can be visualised and reduced to a single representative feature that is the most informative in the cluster (21, 87). Unsupervised data-driven algorithms that project the feature space into a lower dimension can also be used to select non-redundant feature subsets. Principal Component Analysis (PCA), which is a commonly employed method (28, 91, 92), is a linear dimensionality reduction technique that identifies successive axes that account for the largest variance in the data and projects the original feature space onto the hyperplane defined by those axes. The new, dimension reduced feature space is comprised of the principal components, each of which is defined by the projection of the original data onto one of the principal axes. The orthogonality of the principal components ensures that feature collinearity is minimised, which makes it an advantageous technique for the unsupervised removal of redundant features (28). Non-linear dimensionality reduction techniques such as local linear embedding (LLE) can also be used for the selection of salient features (84). It should be noted, however, that the projection of features into a lower dimensional space comes at the cost of reduced feature interpretability.

### 2.6 Model Development

Having selected a subset of salient features, the final step in the radiomics workflow is the building of the predictive model. The development of classification models can be done using a variety of ML and deep learning techniques. It is impossible to know *a priori* which method will generate the best predictive model, and thus experimentation is advised along with comprehensive documentation of the techniques tested, hyperparameters used, and validation results. Scientific reproducibility of radiomics studies is a critical factor that can be facilitated by making implemented code available on platforms such as Github^1^. A summary of some of the more prominent modelling techniques used, and example papers where they have been used in relation to mPCa, are provided in **Table 1**.

**Table 1:** Summary of the prominent modelling techniques used in radiomics research.

Category	mPCa Papers	Technique	Description	Advantages	Disadvantages
Machine Learning	(93)	Support Vector Machine	Determines the support vectors in multidimensional space that define the hyperplane best separating the classes	Versatile algorithm capable of both linear and non-linear classification	Not suited to large datasets Model is difficult to interpret Sensitive to feature scaling
	(94, 95)	Logistic Regression	Binary classification algorithm	Probabilistic output Relatively quick training time	Linear assumption makes it unsuitable for highly complex datasets
	(96)	K- Nearest Neighbour	Classifies new instances based on their calculated distance from the k-nearest neighbours in the memorised training set.	Instance-based model – no training period	Does not scale well to large datasets Sensitive to noise, outliers and feature scale
	(96)	Decision Tree	Hierarchical model structure resembling a tree where instances are partitioned at nodes based on feature threshold values until a final classification is made at the leaf nodes	Highly interpretable – ‘white box’ classification method Non-parametric model - makes limited assumptions about training data Insensitive to feature scale	Prone to overfitting Small changes in training data can cause large changes to tree structure, leading to model instability.
	(97, 98)	Ensemble-Based Methods (Random Forest, Stacked Generalisation, Bootstrapping Aggregation, etc.)	Aggregates predictions from multiple trained classifiers and combines them into a single predictive model	Can combine several weak classifiers into a single strong classifier Reduces prediction model bias	Very limited model interpretability Depending on the base classifiers used, extensive hyperparameter tuning may be required.
Deep Learning	(99)	Convolutional Neural Network	Repeated application of convolution operations to an input vector/image to learn salient feature maps for the desired classification task.	Capable of performing end-to-end feature extraction and predictive modelling Highly versatile Automatically learns important image features without the need for hand-crafted feature engineering	Potentially limitless number of possible architectures with limited *a priori* knowledge of which is best suited to the task Typically require large datasets to be generalisable Large training time Severely limited model interpretability
	(82, 100)	Transfer Learning	Extracting layers in deep networks that have been pre-trained on large datasets to be used as feature extractors for your specific task	Useful for small dataset problems where there are insufficient samples to train a full deep network Can decrease training time significantly	Can fail if the outcome of the pre-trained network differs significantly from that of the target outcome. Severely limited model interpretability

Exemplar papers using each of these in mPCa radiomics studies are provided.

## 3 Conventional mPCa Biomarkers and Risk Factors – A Brief Overview

Various patient biomarkers and characteristics are factored into assessing the risk of developing mPCa and the clinical management of the disease if it subsequently develops. Patient characteristics such as age, presence of co-morbidities, previous treatment history and personal preference can all affect mPCa management (101). PSA concentrations carry prognostic information, with elevated levels associated with an increased risk of metastatic development (102). Changes between pre- and post-therapeutic PSA levels is also used to assess patient response to treatment (103), however, the non-specificity of PSA to PCa raises questions about its usefulness in this respect (11). Gleason grading is also a powerful prognostic tool, both for localised PCa and mPCa in either the castrate-resistant form or the castrate-sensitive form (104–106). To inform a precision medicine approach to mPCa management, numerous molecular assays exist that can provide prognostic information. Detecting the presence of the AR-V7 splice variant in circulating tumour cells, for example, is a factor predictive of poor therapeutic response to androgen receptor inhibitors such as enzalutamide and abiraterone (107).

In addition to clinicopathologic characteristics, first-order radiomics imaging features have also demonstrated usefulness in the clinical management of mPCa. Standardised uptake value (SUV)_max_ of identified tumours is a prognostic imaging biomarker (108), and when measured from [^18^F]-fluorodeoxyglucose (FDG) PET-avid lesions has been shown to correlate with patient survival (109). Total lesion glycolysis (TLG) or total SUV (SUV_total_), measured as the sum of individual SUVs in each voxel for each individual lesion, is another prognostic imaging biomarker that has been shown to correlate with overall survival in metastatic castration-resistant prostate cancer (mCRPC) patients (110). Radiomics possesses great potential because rather than attempting to replace these biomarkers, clinicopathologic risk factors and patient characteristics can be incorporated into the modelling process and leveraged to make better model predictions.

## 4 Review Methodology

### 4.1 Study Inclusion Criteria

**Figure 2:**
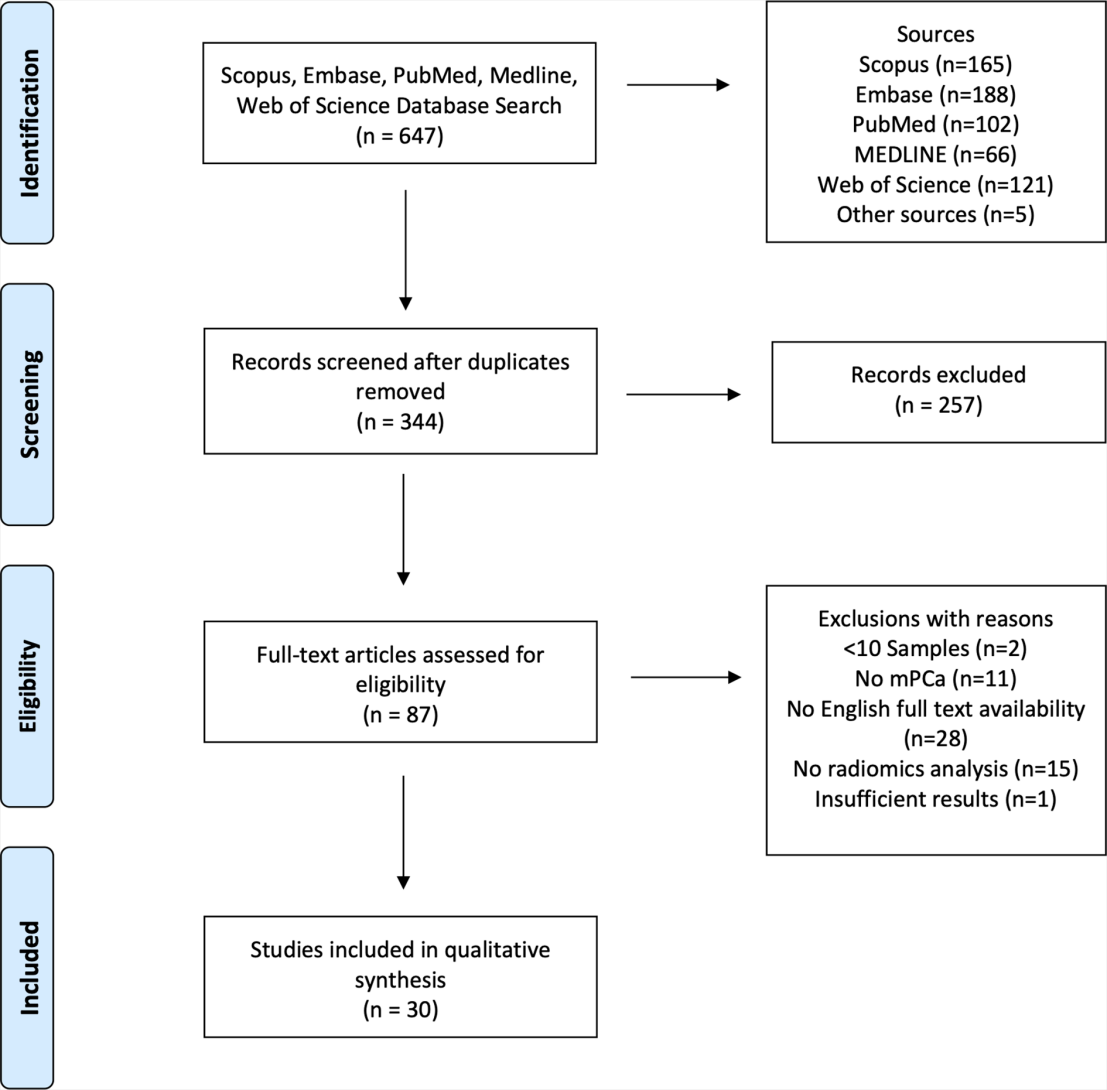
PRISMA flow diagram showing the study inclusion and exclusion process.

Databases were searched using a logical search string [(“radiomics” OR “texture analysis” OR “radiological features” OR “radiomic features” OR “textural features” OR “texture features” OR “deep learning” OR “machine learning” OR “convolutional neural network” OR “CNN”) AND (metastatic OR metastases) AND (“prostate cancer” OR “prostate lesions”)] to identify potentially relevant papers published before the 23^rd^ of June 2021. Inclusion criteria were as follows: (1) human studies only, (2) analysis of medical imaging modalities only (radiomics analyses of histopathology slides were not included), (3) papers must either (i) extract a minimum of second order statistical features, or (ii) use deep learning techniques for feature extraction, (4) results must have diagnostic, prognostic, or predictive applicability to mPCa, (5) minimum of 10 sample size, and (6) full text of article must be available and accessible through our institution. A flow diagram illustrating the inclusion of identified studies is provided in the form of a PRISMA diagram (111) in **Figure 2**.

The studies were partitioned into two sections following their inclusion into this review, namely, (i) Traditional radiomics, referring to papers that utilised traditional hand-crafted feature extraction techniques, and (ii) Deep radiomics, referring to papers that utilised deep learning networks for the extraction of deep features.

### 4.2 RQS Criteria

Identified papers utilising traditional hand-crafted feature extraction were subject to a RQS analysis. The RQS criteria is comprised of 16 defined items that correspond to critical points in the radiomics workflow. Each item in the RQS either awards or deducts points from a paper according to the study methodology. The RQS is designed to pinpoint methodological weaknesses in radiomics studies and encourage scientifically rigorous radiomics investigations (32). **Table 2** provides a full description of these items and the points that can potentially be gained at each. A study may achieve a maximum of 36 points, or a minimum of -8 points. Each paper was assigned a percentage based on their score out of 36.

**Table 2:** Details of RQS items and associated points awarded/deducted at each step.

RQS Item	RQS Item Name	Item Description (points awarded/subtracted)
1	Image protocol quality	Well documented image protocols (+1) Public protocol used (+1)
2	Multiple Segmentations	Feature robustness testing to segmentation variabilities, e.g. different algorithms or physicians (+1)
3	Phantom study on all scanners	Feature robustness testing to scanner parameters (+1)
4	Multiple time points	Feature robustness testing to temporal variabilities e.g. organ movement/shrinkage (+1)
5	Feature reduction	Perform feature reduction or adjust for multiple testing (+3); otherwise (-3)
6	Non-radiomics feature inclusion	Model includes non-radiomic variables/features (+1)
7	Detect biological correlates	Detect and discuss biological correlates (+1)
8	Cut-off analysis	Determine risk groups by either the median, a previously published cut-off value or present a continuous risk variable (+1)
9	Discrimination statistics	Report a discrimination statistic and its statistical significance (+1) A resampling method is also applied (+1)
10	Calibration statistics	Report a calibration statistic and its statistical significance (+1) A resampling method is also applied (+1)
11	Prospective study	Prospective methodology to validate a radiomics signature (+7)
12	Validation	No validation (-5) Internal validation (+2) External validation from one institute (+3) External validation from two institutes (+4) Validating a previously published radiomics signature (+4) External validation from three or more institutes (+5)
13	Gold standard comparison	Assess model agreement/superiority to current ‘gold standard’ (+2)
14	Clinical utility	Quantify model applicability in clinical setting e.g. decision curve analysis (+2)
15	Cost-effectiveness assessment	Assess cost-effectiveness of radiomics signature (+2)
16	Open science and data	Scans are open source (+1) Region of interest segmentations are open source (+1) Code is open source (+1) Features are calculated on an open-source set of representative features (+1)

## 5 Radiomics in mPCa – Results

### 5.1 Traditional Radiomics

The use of the traditional radiomics paradigm of utilising hand-crafted features in mPCa is the predominant method of solving clinical challenges. **Tables 3**, **4** summarise the salient characteristics for all identified traditional radiomics papers.

**Table 3:** Prominent characteristics of identified mPCa studies using traditional radiomics methods with PET and/or CT modalities.

First Author	Year	Imaging Series Analysed	Design	Patient sample size	Patient Cohort	Radiomics Software	Outcome Measures	Clinical Covariates Included	External Validation	Results Synopsis	RQS (%)
Acar E (96)	2019	CT	R	75	PCa patients with metastatic bone disease	LifeX	Sclerotic Bone Lesion Response	N	N	Acc = 73.5%, AUC = 76.0% Sens = 73.5%, Spec = 73.7%	0
Alongi P (112)	2021	^18^F-Choline PET	R	94	High Risk PCa	LifeX	LNI, Distant Metastasis	Y	N	LNI AUC = 69.87 (95% CI 51.34 - 88.39) Distant Metastasis AUC = 74.72 (95% CI 56.36 - 93.09)	27.78
Cysouw M.C.F (97)	2020	^18^F-DCFPyL PET	P	76	Intermediate- to high-risk primary PCa	RaCaT	LNI, Distant Metastasis	N	N	LNI AUC = 0.86 ± 0.15, p < 0.01 Distant Metastasis AUC = 0.86 ± 0.14, p < 0.01	47.22
Hayakawa T (113)	2020	CT	R	69	PCa patients with pelvic bone metastases	3D Slicer	OS, CSS	Y	N	*Maximum 2D Diameter* and *Least Axis* were risk factors for OS (HR = 1.007 & HR = 1.013, respectively) No features were risk factors of CSS.	0
Khurshid Z (103)	2018	^68^Ga-PSMA PET	R	70	mCRPC patients planned to undergo ^177^Lu-PSMA therapy	NS	Correlation with treatment response	N	N	*Entropy* and *Homogeneity* correlated with response (r = -0.327 & *r* = 0.315, respectively)	8.33
Lin C (89)	2019	^18^F-NaF PET	P	14	mCRPC patients undergoing androgen therapy.	NS	R/R	N	N	*Skewness*, *Kurtosis* and *diagonal moment* exhibited greater R/R than SUV_max_	5.56
Moazemi S (114)	2020	^68^Ga-PSMA PET/CT	R	72	Histologically confirmed PCa	InterView FUSION Software	Hotspot classification	N	N	AUC = 0.98, Sens = 0.94 Spec = 0.89	5.56
Moazemi S (115)	2021	^68^Ga-PSMA PET/CT	R	83	Advanced PCa undergoing ^177^Lu-PSMA therapy.	NS	OS	Y	N	*PET Kurtosis & SUV_min_* significantly correlated with OS	0
Peeken J (116)	2020	CT	R	80	Recurrent PCa	PyRadiomics	LNI	N	Y	AUC = 0.95 (95% CI 0.88 – 0.99)	47.22
Perk T (98)	2018	^18^F-NaF PET/CT	R	37	mCRPC patients	NS	Bone Lesion Classification	Y	N	AUC = 0.95 (95% CI 0.93 – 0.96)	25
Zamboglou C (117)	2019	^68^Ga-PSMA PET	Both	P (n=20) R (n=40)	Histopathologically proven primary adenocarcinoma	In-house MATLAB software	LNI	N	N	Prospective Cohort AUC = 0.87 Retrospective Cohort AUC = 0.85	52.78

Acc, Accuracy; AUC, Area Under Curve; CSS, Cause-specific survival; HR, Hazard Ratio; NS, Not Specified; OS, Overall Survival; P, Prospective; R, Retrospective; R/R, Response-to-repeatability; Sens, Sensitivity; Spec, Specificity.

**Table 4:** Prominent characteristics of identified mPCa studies using traditional radiomics methods with MRI modalities.

First Author	Year	Imaging Series Analysed	Design	Patient sample size	Patient Cohort	Radiomics Software	Outcome Measures	Clinical Covariates Included	External Validation	Results Synopsis	RQS (%)
Damascelli A (93)	2021	T2w, ADC	R	62	Biopsy confirmed PCa who underwent RP	3D Slicer	LNI	N	N	Acc = 0.9 ± 0.04 Sens = 0.9 ± 0.01 Spec = 0.9 ± 0.01	25
Hou Y (82)	2021	T2w, DWI, ADC	R	401	Biopsy confirmed PCa patients who underwent RP & ePLND	PyRadiomics	Pelvic Lymph Node Metastases	Y	Y	AUC = 0.76 (95% CI, 0.62-0.87)	33.33
Li L (118)	2021	T2w, ADC	R	198	PCa patients who underwent RP	NS	LNI	Y	Y	AUC = 0.77 (95% CI, 0.67-0.86)	50
Reischauer C (119)	2018	ADC	P	12	Treatment-naïve advanced PCa with scintigram confirmed metastases	In-house MATLAB software	Change in response to ADT therapy	N	N	Numerous first- and second-order statistical features showed promise in monitoring treatment response and correlation to changes in serum PSA levels over time.	0
Wang Y (94)	2019	T2w, DCE T1w	R	176	Histologically confirmed PCa without evidence of distant metastasis	IBEX	Prediction of Bone Metastasis	Y	N	AUC = 0.895 (95% CI 0.836 - 0.939)	25
Zhang W (95)	2020	T2w, DCE T1w, DWI	R	116	Biopsy confirmed PCa	AK Software	Bone Metastases	Y	N	AUC = 0.93 (95% CI, 0.86 – 0.99)	38.89

Acc, Accuracy; ADT, Androgen deprivation therapy; AUC, Area Under Curve; ePLND, extended pelvic lymph node dissection; NS, Not Specified; OS, Overall Survival; P, Prospective; R, Retrospective; Sens, Sensitivity; Spec, Specificity.

#### 5.1.1 Metastasis Prediction and Detection

The prediction of metastases development, or the detection of its presence, are the most common outcome objectives of the traditional radiomics studies identified in this review. Wang et al. (94) constructed a prognostic model for the pre-treatment prediction of bone metastases in patients with histologically confirmed PCa without evidence of distant metastatic spread. 976 radiomic features, including first-order statistics, shape features and higher order texture features, were extracted from the outlined primary PCa lesion on T2-weighted (T2w) and dynamic contrast-enhanced (DCE) T1-weighted (T1w) images. The final logistic regression model, comprised of imaging features weighted by their least absolute shrinkage and selection operator (LASSO) regression coefficients and clinicopathologic patient variables (age, GS, free PSA) achieved good discriminative performance in predicting future bone metastases in the internal validation cohort, with an AUC of 0.895 (95% CI 0.836 - 0.939). The radiomics model outperformed traditional prognostication methods, such as the GS (AUC = 0.731), demonstrating the potential for non-invasive assessment of primary PCa tumours on mp-MRI for prediction of future bone metastases, with potentially profound impacts for individualised patient therapeutic decision making. These results are in good agreement with those achieved by Zhang and colleagues (95) who developed a combined radiomics and clinicopathologic variable (total PSA) nomogram for assessing the risk of bone metastases in newly diagnosed PCa patients from features extracted from T2w, DCE T1w and diffusion-weighted imaging (DWI) scans. LASSO regression was used to perform feature selection and generate the radiomics score, where each feature was weighted by its regression coefficient, and then combined with the total PSA clinical variable in a multivariable logistic regression analysis. The final nomogram achieved good predictive performance in an internal validation cohort (n = 35) in the prediction of developing bone metastases with an AUC = 0.93 (95% CI, 0.86 – 0.99).

PET imaging features can be highly effective in the detection of PCa metastases. Cysouw et al. (97) conducted a study on a cohort of 76 intermediate- to high-risk primary PCa patients who were scheduled to undergo radical prostatectomy, where radiomics features were extracted from the delineated intraprostatic tumour volume to develop a diagnostic model for detecting the presence of either lymph node metastases or any distant metastases in [^18^F]DCFPyL PET scans. Intensity, shape-based and texture features were extracted from the delineated intraprostatic primary PCa lesion and subject to ML analysis. The resulting random forest algorithm trained on a subset of features chosen *via* three different feature selection methods (PCA, recursive feature elimination with random forest, and univariate analysis of variance) and utilising 5-fold cross validation achieved good discriminatory performance in the detection of LNI (AUC = 0.86 ± 0.15, *p* < 0.01), and any distant metastasis (AUC = 0.86 ± 0.14, *p* < 0.01). Feature importance analysis of the random forest ML algorithm determined that two intensity-based features, *difference volume at intensity fraction* and *volume at intensity fraction 10*, were the most important in detecting LNI and distant metastases (both had importance coefficients of 0.11). The clinical relevance of the ML model is that it can non-invasively aid in the determination of low-risk patients that can be spared extended pelvic lymph node dissection and its associated complications (120).

A similar study was conducted by Zamboglou et al. (117), who enrolled both a prospective (n=20) and a retrospective (n=40) validation cohort of patients with intermediate- to high-risk PCa who underwent ^68^Ga-PSMA PET/CT imaging prior to RP. Intraprostatic lesions in the PSMA PET image in the prospective cohort were segmented in three ways; (i) by histopathologic analysis and *ex-vivo* CT scanning of the RP specimen, which was subsequently transferred to the *in-vivo* CT scan before being transformed into the PSMA PET coordinates; (ii) manually by two nuclear medicine physicians, and; (iii) semi-automatically by the application of local 40% thresholds in the intraprostatic volume. The retrospective validation cohort was segmented only manually. In the prospective cohort, the QSZHGE (quantised short zones high-gray level emphasis), which was robust to different scanner parameters (determined in a phantom study) and to the three segmentation methods, was able to discriminate well between patients with and without LNI (AUC = 0.87 for the expert manual segmentation, and AUC = 0.85 for the histopathologic segmentation, both *p* < 0.01). This performance was validated in the retrospective cohort (AUC = 0.85, *p* < 0.01). Alongi et al. (112) analysed 94 high-risk PCa patients who underwent ^18^F-Choline PET/CT restaging imaging to determine features predictive of disease progression. Follow up data was recorded for the patients for a median of 26 months (range 13-52) and subsequent TNM staging performed. Discriminant analysis on the radiomics features extracted yielded a machine learning (ML) model capable of achieving moderate predictive power in the development of nodal (AUC = 69.87, 95% CI 51.34–88.39) or distant metastases (AUC = 74.72, 95% CI 56.36–93.09). *HISTO entropy log10* and *HISTO entropy log2* were the two salient features chosen for the discrimination of distant metastases, while *GLSZM SZLGE* and *HISTO energy uniformity* were the chosen features to predict nodal metastases.

Studies have also shown the great potential of radiomics features extracted from mpMRI modalities in the detection and prediction of metastases. Damascelli et al. (93) developed a radiomics model for the prediction of LNI on 62 patient mpMRI scans, where features were extracted from T2w images and apparent diffusion coefficient (ADC) maps. Each patient, who had biopsy proven PCa and underwent RP, had their intraprostatic index lesions segmented on each modality by two independent radiologists. Features not robust to the two segmentations (Friedman test *p* value > 0.01) were excluded, and the covariance matrix analysed to remove redundant features. Support vector machine classifiers were built using features from each modality both separately and in combination, and were able to predict lymph node metastasis presence with good accuracy (ADC alone, Acc = 0.86 ± 0.05; T2w alone, Acc = 0.84 ± 0.05; ADC + T2w features, Acc = 0.9 ± 0.04). Their results demonstrate the promise of utilising radiomics signatures derived from mpMRI scans to predict LNI and the potential of utilising features extracted from multiple modalities in radiomics predictive modelling to capture complementary information about lesions and improve overall model performance. Li et al. (118) performed a comparable analysis and developed a prognostic radiomics nomogram for the pre-operative prediction of lymph node metastases also in PCa patients who underwent RP. A total of 200 radiomic features were extracted from the intraprostatic index lesion delineated in both the T2w image and the ADC maps, where non-repeatable features were excluded by performing a test-retest analysis on an openly available test-retest mpMRI dataset (121), and the remaining features were selected using a 5-fold 10-run cross validation of a minimum redundancy maximum relevance algorithm for Cox-proportional hazards model building. This model was combined with clinicopathologic patient data (PSA and GS) to generate the final prognostic nomogram, which was externally validated on a multi-institutional dataset. Validation set performance in the prediction of LNI was AUC = 0.77 (95% CI, 0.67-0.86), and this result compared favourably to other prognostic tools used for the prediction of post-treatment adverse pathology such as the Prostate Cancer Risk Assessment score (AUC = 0.74; 95% CI, 0.62-0.85) and the Decipher risk score (AUC = 0.73; 95% CI, 0.59-0.87). The multi-institutional validation and superior performance of this nomogram compared to other prognostic tools is strong evidence for the potential of utilising radiomics features for the pre-operative risk stratification of patients.

Hou et al. (82) in a recent study explored how traditional radiomics features can be supplemented with deep learning machine learnt features in the prediction of pelvic lymph node metastases (PLNMs). A relatively large sample size of 401 patients (including a 50-patient external validation set) with biopsy confirmed PCa were analysed, where 2553 radiomics features were extracted in total from the index intraprostatic lesion on mpMRI modalities (T2w, DWI with b = 1500s/mm^2^ and ADC maps) to generate a radiomics signature. In parallel, five pre-trained deep neural networks trained on ImageNet data were utilised in a transfer learning framework which leverages the high-level salient feature extraction ability of the pre-trained networks and applies them to the present problem. Random forest algorithms were used to output a final risk score reflecting the probability of the patient developing pelvic lymph node metastatic disease for both the radiomics and the deep learning signatures combined and individually. The final risk model yielded an AUC = 0.76 (95% CI, 0.62-0.87) on the external hold-out set. Furthermore, the authors compared their risk model with established prognostic tools such as the Memorial Sloan Kettering Cancer Center (MSKCC)^2^ nomogram and the Briganti score (122), determining that an optimal threshold of 8% on their risk model is superior to both in terms of sparing unnecessary pelvic lymph node dissections and missing fewer true positive PLNMs.

#### 5.1.2 Lesion Classification

Radiomics features have also been used successfully to classify the malignancy of segmented lesions, for example, Peeken et al. (116) developed and externally validated a radiomics signature extracted from contrast-enhanced CT images to determine the malignancy (positive or negative) of segmented lymph nodes. A total of 149 lymph nodes were segmented from which shape, first-order, textural, and local binary pattern-based intensity features were extracted. LASSO regression was used to select salient features for the final model, which performed well on a dedicated external validation cohort (n = 33 patients) with an AUC = 0.95 (95% CI 0.88 – 0.99). Their results demonstrate that radiomics feature extraction can be extended to ROIs other than the tumour volume, such as segmented lymph nodes, and still yield accurate predictive models. Additionally, the authors of this study also utilised ComBat batch harmonisation to correct for the difference in scanner parameters between the external validation cohort and the main training cohort, but found that the use of this technique did not significantly change the results of the radiomics model.

Similar lesion classification analysis has been conducted on other imaging modalities. Moazemi et al. (114) employed ML analysis on radiomics features to classify 2419 ^68^Ga-PSMA PET hotspots (defined as uptake above the background concentration) in 72 patients as either benign or malignant. A total of 80 features were extracted from each hotspot (40 from the PET image and 40 from the CT image) and utilised in five different ML algorithms, with the Extra Trees model achieving exemplary discriminatory performance in the classification of lesions in the hold-out validation set (n = 24) with AUC = 0.98 (Sens = 94%, Spec = 89%). Their results also further demonstrate the validity of multi-modal analysis since features from the CT and PET images used together in the ML models slightly outperforms using either of them separately. Perk et al. (98) conducted a similar analysis on ^18^F-NaF PET/CT images in a cohort of 37 mCRPC subjects. Bone lesions were delineated by an automated algorithm that determines the lesion boundaries based on statistically optimised regional thresholding (SORT) which uses a different threshold based on the location of the lesion in the patients skeleton (123), which were subsequently assigned a classification label by a nuclear medicine physician between 0 and 5 depending on the likelihood of malignancy (0; background ROI, 1; Definite Benign, 5; Definite Malignant). Radiomics features were extracted from both the PET and CT images and used as the input for ML analysis with nine separate learning methods, where the random forest model performed the best under 10-fold cross-validation conditions at discriminating between the 0 + 1 vs. 5 class labels (AUC = 0.95, 95% CI 0.93 – 0.96).

#### 5.1.3 Treatment Response

PSMA-labelled isotopes are becoming increasingly important in the imaging and treatment of metastatic PCa. ^177^Lu-PSMA therapy, in particular, is gaining prominence as a radioligand therapeutic intervention for advanced PCa, however, it is known that a large percentage of interventions will not be successful (124). Early identification of which patients may benefit from a particular intervention type can be substantially aided by radiomics analysis which can yield useful pre-therapeutic biomarkers. Moazemi et al. (115) extracted radiomics features from delineated hotspots (n = 2070) in advanced PCa patients with pre-therapeutic ^68^Ga-PSMA PET/CT imaging. Following a LASSO regression feature selection process, they determined that a radiomics signature containing *PET Kurtosis* and *SUV_min_* were significantly correlated with overall survival (*r* = 0.2765, *p* =0.0114). An earlier study by Khurshid et al. (103) performed a similar retrospective analysis on 70 mCRPC patients scheduled to undergo ^177^Lu-PSMA therapy. Metastatic lesions in each patient (total ROI’s = 328) were segmented, from which histogram and textural features from the normalised gray-level co-occurrence matrix (NGLCM) were extracted. Therapeutic response was defined according to the change in pre- and post-therapy PSA levels, which is common practice, although, as an aside, recent evidence has demonstrated the potential for texture features to be used as biomarkers for therapeutic response assessment (119). Their analysis showed that two textural parameters extracted from the NGLCM of bone lesions correlated with the change in PSA levels following therapy, these being *entropy* (*r* = -0.327, *p* = 0.006) and *homogeneity* (*r* = 0.315, *p* < 0.008). The results therefore indicated that the lesions which were more heterogeneous in nature responded better to the ^177^Lu-PSMA therapy. This is an interesting result that can potentially inform future clinical decision making regarding the use of ^177^Lu-PSMA radioligand therapy, pending future prospective and external validation.

Determining the status of sclerotic metastatic lesions post-treatment is difficult because even responded lesions that are no longer metastatic can retain their sclerotic character when pre-treatment and post-treatment CT images are compared. Acar et al. (96) sought to utilise radiomics imaging features to discriminate between sclerotic lesions that were completely responded or still metastatic following therapeutic interventions. Histogram, shape-based, and texture features were extracted from manually delineated sclerotic bone lesions in the non-contrast CT scan of each patient. Sclerotic lesions were categorised as completely responded or metastatic if they had ^68^Ga-PSMA PET uptake levels either below or above the measured liver expression. Multiple ML models were developed, including a weighted K-nearest neighbour (KNN), support vector machine, decision tree and ensemble-based methods, where the weighted KNN achieved the best classification performance under 10-fold cross-validation conditions (Accuracy = 73.5%, AUC = 76.0%, Sensitivity = 73.5%, Specificity = 73.7%). The potential for non-invasive sclerotic bone lesion response assessment using non-contrast CT imaging is demonstrated in this study, however, the lack of external validation, retrospective design, and limited patient sample size (n = 75) indicate that further studies are necessary.

#### 5.1.4 RQS Assessment

Of the papers identified in this review that used traditional hand-crafted features, the mean RQS was 23% ± 19.6% (range: 0 – 52.8%). This low score is the continuation of a trend in radiomics research, where overall low methodological quality has been identified with respect to the RQS by a number of different authors across a variety of radiomics use cases (34–38). Only 17.6% of studies conducted an external validation of their results (3/17) and only four papers were prospective in nature or had at least a prospective component (4/17, 23.5%). Assessment of feature reproducibility was in general low. Seven studies (7/17, 41.1%) performed multiple segmentations to assess robustness of features to each, but only a single paper conducted a phantom study to assess the robustness of features to the scanner variabilities (1/17, 5.9%). Other major limitations identified include: failure to include a calibration statistic and its statistical significance (15/17, 88.2%); the overwhelming lack of accordance to open scientific principles (16/17, 94.1%); no detection of biological correlates (0/17); and no cost-effectiveness analysis (0/17). The majority of studies did undertake some form of feature reduction to reduce the chances of overfitting (12/17, 70.6%), and just under half of identified papers included non-radiomics features (such as clinical covariates) into their model building process (8/17, 47.1%). **Supplementary Table 1** shows how the studies fared with respect to each RQS item.

### 5.2 Deep Radiomics

Utilising deep learning for automated end-to-end salient feature learning, extraction and modelling (which we may term ‘Deep Radiomics’) instead of traditional hand-crafted features is an area of study that has considerable potential. The rise of the field has been facilitated by an overall increase in the availability of computational resources and toolkits^3,^ ^4,^ ^5^ that have made the designing and training of these networks easier than ever before. The potential of these models to become crucial pieces of the clinical workflow, supplementing physician decision making and contributing to individualised patient therapy, is enormous. In the deep radiomics publications identified in this review, the detection or classification of patient metastatic lesions is the overwhelming outcome measure.

With respect to imaging modalities, bone scintigraphy was the modality that was most commonly analysed (100, 125–131), and the salient characteristics of these papers are summarised in **Table 5**. Papandrianos and colleagues (128) designed a CNN architecture for bone metastases diagnosis, where patient bone scintigrams were classified into three classes: no metastasis, degenerative (defined as the absence of metastasis but the presence of degenerative lesions), and metastasis present. Of the 778 patient bone scans examined 15% were reserved solely for testing and following a thorough exploration of the optimal CNN hyperparameters to be used, the final architecture achieved overall classification accuracy of 91.61% ± 2.46% (F1-score = 0.938). This result concords with a very similar study undertaken by the same authors, except in this instance only performing a dual-class classification problem (metastasis present vs. metastasis absent) by excluding any patients with degenerative lesions where their CNN model achieved a higher overall accuracy of 97.38% (129). Ntakolia et al. (127) performed the same three-class classification problem mentioned above also on 778 PCa patients who underwent bone scintigraphy, except this time deploying a lightweight version of the look-behind FCN (LB-FCN) (132, 133) and achieved a better overall accuracy of 97.41%. Their results demonstrated that state-of-the-art classification results can be achieved using a CNN with less learnable parameters and thus requiring less resources for training.

**Table 5:** Prominent characteristics of identified mPCa studies using deep radiomics methodology on bone scintigrams.

First Author	Year	Design	Patient sample size	Patient Cohort	Deep Network Architecture	Outcome Measures	External Validation	Results Synopsis
Aoki Y (125)	2020	R	139	PCa	Fused dual U-Net	Bone metastases	N	Acc = 89.2% Sens = 91.7% Spec = 87.3%
Cheng D (100)	2021	R	194	PCa	YOLO v4	Chest hotspot classification	N	Sens = 0.72 ± 0.04 Prec = 0.90 ± 0.04
Cheng D (126)	2021	R	205	PCa	R-CNN & YOLO v3	Chest & Pelvic hotspot classification	N	Chest Classification Sens = 0.82 ± 0.08 Prec = 0.70 ± 0.11 Pelvis Classification Sens = 0.87 ± 0.12 Prec = 0.81 ± 0.11
Ntakolia C (127)	2020	R	778	PCa	LB-FCN *light*	Bone metastasis diagnosis	N	Overall accuracy = 97.41%
Papandrianos N (128)	2020	R	778	PCa	CNN	Bone Metastasis Diagnosis	N	Overall accuracy = 91.61% ± 2.46%
Papandrianos N (129)	2020	R	586	PCa	CNN	Bone Metastasis Diagnosis	N	Overall accuracy = 97.38% Sens = 95.8%
Sadik M (130)	2008	R	614	PCa patients with suspected metastatic disease	ANN	Bone Metastasis Diagnosis	N	Sens = 90% Spec = 89%
Wuestemann J (131)	2020	R	149	PCa patients with malignant indication on bone scan	ANN	Bone Metastasis Diagnosis	Y	AUC = 0.937, Sens = 87.0% Spec = 98.6%, PPV = 98.5% NPV = 87.7%

Acc, Accuracy; AUC, Area Under Curve; NPV, Negative Predictive Value; P, Prospective; PPV, Positive Predictive Value; R, Retrospective; Sens, Sensitivity; Spec, Specificity.

Deep learning techniques can also be utilised to classify individual identified lesions, rather than whole patient images, in bone scintigrams. Cheng et al. (100) used data from 371 breast cancer patients to pre-train a YOLO v4 network (134) to classify identified chest hotspots in bone scintigraphy images as either metastatic or benign. Employing a transfer learning approach, the model was then trained on a dataset of 194 PCa patients under a 10-fold cross-validation scheme which yielded a lesion-level classification sensitivity of 0.72 ± 0.04 and a precision of 0.9 ± 0.09. Their approach suggests the feasibility of utilising metastatic lesions in malignancies other than prostate cancer for pre-training a classification network, thus potentially offering a way for researchers to generate functioning networks even with limited datasets. A follow-up study (126) using instead the YOLO v3 network (135) managed to increase the sensitivity (0.82 ± 0.08) at the expense of precision (0.7 ± 0.11) for chest hotspot classification.

The remaining identified studies investigated deep learning on PET and/or CT images with various labelled radiotracers (99, 136–139). **Table 6** shows the important characteristics of these studies. Lee et al. examined a cohort of 251 PCa patients with suspected BCR following definitive primary therapy (139). ^18^F-Fluciclovine PET images were labelled as either ‘normal’, if no recurrence was found, or ‘abnormal’, if there was either a recurrence in the prostatic bed or evidence of pelvic metastasis. Two 2D CNN’s (ResNet-50) were trained (a slice-based model which predicts individual patient PET slices, and a case-based model that makes a prediction for the entire PET image), and one case-based 3D CNN (ResNet-14) was trained to predict image abnormality. Prediction results on a dedicated test set at the patient level (AUC = 0.75, *p* = 0.013; Sens = 85.7%; Spec = 71.4%) were outperformed by the 2D slice-based CNN (AUC = 0.971, *p* < 0.001; Sens = 90.7%; Spec = 95.1%), however, both 2D models outperformed the 3D ResNet-14 (AUC = 0.699, *p* = 0.053; Sens = 71.4%; Spec = 71.4%). The authors hypothesise that the reason for the underperformance of the 3D CNN could be due to the fact that the 3D CNN had a higher number of learnable parameters and would therefore require a greater training dataset size to generate a sufficiently generalisable model.

**Table 6:** Prominent characteristics of identified mPCa papers using deep radiomics methodology on PET and/or CT modalities.

First Author	Year	Imaging Series Analysed	Design	Patient sample size	Patient Cohort	Deep Network Architecture	Outcome Measures	External Validation	Results Synopsis
Borrelli P (136)	2020	^18^F-Choline PET/CT	R	399	Biopsy-proven PCa	Dual CNN architecture for detection & segmentation	Lymph node metastases detection, association with survival	N	The number of metastatic lymph nodes detected is associated with PCa-specific survival (HR = 1.19, 95% CI 1.05 – 1.33).
Lee J (139)	2020	^18^F-fluciclovine PET	R	251	PCa patients with suspected BCR following definitive therapy	ResNet-50 & ResNet-14	Pelvic recurrence or metastasis classification	N	Patient-level predictions AUC = 0.750 (*p* = 0.013) Sens = 85.7% Spec = 71.4% Slice-level predictions AUC = 0.971 (*p* < 0.001) Sens = 90.7% Spec = 95.1%
Hartenstein A (137)	2020	CT	R	549	Histologically confirmed PCa	CNN	LNI	N	AUC = 0.95 Sens = 86% Acc = 89% Spec = 92%
Masoudi S (138)	2021	CT	R	114	Metastatic PCa (41 histologically confirmed)	2D ResNet-50 & 3D ResNet-18 Ensemble	Bone lesion classification	N	Acc = 92.2% F1 Score = 92.3%
Zhao Y (99)	2019	^68^Ga-PSMA PET/CT,	R	193	mCRPC	2.5D U-Net Ensemle	Pelvic area metastatic lesion detection	N	Precision range 0.79 – 0.99 depending on the type of lesion detect (bone, lymph node, or local) Sensitivity range 0.61 – 0.99

Acc, Accuracy; AUC, Area Under Curve; HR, Hazard Ratio; P, Prospective; R, Retrospective; Sens, Sensitivity; Spec, Specificity.

One can take this analysis further by classifying individual lesions as benign or malignant. Masoudi et al. (138) demonstrated how deep learning can be used to classify the malignancy of individual bone lesions using only a patient staging CT. An expert radiologist identified, annotated, and segmented 2,880 bone lesions in 114 PCa patients, 41 of which were histopathologically confirmed to be metastatic. Fifteen patients were reserved solely for testing purposes. Using a constructed ensemble model comprised of the 2D ResNet-50 and a 3D ResNet-18 architecture, they achieved high accuracy in classifying bone lesions in the test set (Acc = 92.2%). The use of CT to classify the malignancy of patient lesions is not limited exclusively to bone lesions. Hartenstein et al. (137) trained a CNN for the binary classification of lymph nodes in contrast-enhanced CT images of 549 histologically confirmed PCa patients (2,616 labelled lymph nodes identified on ^68^Ga-PSMA PET) and achieved a great accuracy of 89% (AUC = 0.95; Sens = 86%; Spec = 92%). External validation of these results was lacking, however, and the authors acknowledge that future studies should utilise histopathology confirmed lymph node invasion *via* extended pelvic lymph node dissection, rather than ^68^Ga-PSMA PET, as the ground truth reference.

Deep learning can also be applied to perform fully automated detection of metastatic PCa lesions. Automated detection of metastases in patients can relieve some of the significant clinical time burden associated with manual observer analysis of medical images. Zhao et al. (99) conducted a proof-of-concept study for the automated detection and segmentation of metastatic lesions in ^68^Ga-PSMA PET/CT images of mCRPC patients. The authors applied a 2.5D U-Net ensemble network that leveraged information from each anatomical plane separately to make predictions on the presence or absence of metastases in individual voxels, with manual delineations by expert physicians serving as the reference ground truth. Their results, which were restricted only to detecting metastatic disease in the pelvic area, demonstrated great accuracy in the detection of bone lesions (Sens = 0.99, Prec = 0.99) and lymph node lesions (Sens = 0.9, Prec = 0.94), but inferior accuracy in the detection of local prostate lesions (Sens = 0.61, Prec = 0.79). Although highly promising, extending this analysis to detect metastases throughout the body that aren’t restricted to the pelvic area remains a fertile pathway of future research. On top of the clear clinical benefit of having lesions detected automatically, there is potentially valuable prognostic information to be gained from the number of automatically detected lesions. Borrelli et al. (136) trained and tested two CNN’s, one that detected important patient organs, and another that used this output in conjunction with the patient ^18^F-Choline PET/CT image to automatically detect lymph node lesions in 399 biopsy-proven PCa patients. They determined that the number of automatically detected lymph node metastases was significantly associated with PCa-specific survival (HR = 1.19, 95% CI 1.05 – 1.33).

## 6 Discussion and Future Recommendations

### 6.1 Quality of the Reviewed Studies

The RQS assessment of the identified papers reveals some significant limitations in the current landscape of mPCa radiomics research. The overall lack of prospective methodology and external validation of developed models, along with the other limitations presented above, are crucial contributing factors that are impeding the translation of radiomics models from being of purely academic interest to being properly realised clinical models capable of facilitating truly personalised patient interventions based on the specific phenotype of their malignancy. These downsides should be addressed as soon as possible so that the full potential of radiomics can be realised.

Having said this, it should also be noted that the RQS as a measure of methodological quality has its own limitations. For example, it takes insufficient account of the nature of the study and penalises papers perhaps too harshly in some respects, while not penalising sufficiently other aspects such as significant overfitting. For traditional radiomics papers whose purpose is not to develop a radiomics model, for example, Lin et al. (89) and their investigation into a potential new metric for response assessment, overfitting is not a consideration and thus feature reduction is not necessary. Similarly for studies using machine-learnt features for extraction and modelling, some aspects of the RQS might not be applicable. Employing feature cut-off analyses, for example, is unnecessary for a variety of ML models that are commonly used in radiomics research and, as has been pointed out elsewhere (36), might even compromise the interpretability of the final ML model. Furthermore, once you move to the realm of deep learning for feature extraction, where the black box nature of the algorithm makes the features that are extracted extremely difficult (if not impossible) to interpret, other parts of the RQS criteria become hard to meet. It is difficult to expect researchers to analyse the robustness of individual features to scanner variabilities when the features are deep features that are not precisely mathematically defined and very difficult to extract individually from a highly complex deep NN or CNN. Assessing individual feature robustness to segmentation variabilities with deep features is similarly impractical but could also be irrelevant. CNN’s, for example, can perform end-to-end feature extraction and predictive modelling on entire medical images without the need for segmentations in the first instance, which should be taken as a significant benefit since it removes what is known to be a large source of bias in radiomics research (32, 53). It would perhaps make more sense in these cases to assess robustness at the model level, rather than the individual feature level, but this is not what the RQS criteria specifies.

Considering these factors, we did not think it appropriate to perform RQS assessments on papers that employed deep learning from end-to-end, since it would make for an unfair comparison to those papers that utilised traditional hand-crafted features. However, even without conducting a RQS assessment of the identified deep radiomics studies, several clear methodological weaknesses exist among the reviewed studies. External validation was poor, with only a single study (131) conducting a validation of the model performance on an external test set. None of the papers were prospective in nature. The implicit bias present in retrospective studies, and the near complete lack of external validation of developed models is a large hurdle to the translation of these models into actual clinical practice. Whether using traditional mathematically defined features, or automatically learnt deep features, if radiomics is to achieve its full potential, then these downsides will need to be addressed in future research.

Model validation is a critical point that needs to be underscored. New predictive, prognostic or diagnostic models need to be validated in some fashion and performing this validation exclusively on the same data on which the model was trained will provide an inflated assessment of model performance. Internal validation should be performed as a necessary first step, where cross-validation techniques, bootstrapping, or a hold-out test set from the original study sample are used to assess model performance. Models that perform poorly on an internal validation sample are unlikely to generalise well to previously unseen populations (140). Internal validation alone, however, is insufficient. Radiomics studies should undertake, as a minimum, internal validation, but ideally external validation should also be conducted where possible (32, 33). If the purpose is to find robust, informative biomarkers and models that can be utilised in a cross-institutional fashion for diagnostic or prognostic purposes then it is imperative that external validation is also undertaken. External validation of a developed model or an identified biomarker, in which the predictive performance is assessed on a separate data sample, is necessary to understand their capacity to generalise to data other than that on which they were trained or acquired. The published literature reviewed in the present work demonstrate a poor performance when it comes to external validation, which is directly in line with a myriad of other studies that have highlighted the overall lack of external validation that is present in radiomics research pertaining to other malignancies (34, 36, 37). This is an issue that directly impacts the translational capacity of developed models into proper clinical practice, and future studies should seek to address this limitation.

### 6.2 Harmonisation and Standardisation for Reproducibility

The present review has revealed a relative paucity of studies that conducted a feature reproducibility analysis with respect to different segmentation techniques or scanner variabilities. Retrospective or multi-institutional datasets often do not have standardised imaging acquisition and reconstruction protocols. While efforts should be made to obtain a dataset of images with consistent or identical scanner parameters, this is not always feasible. Harmonisation techniques have been employed and validated that limit the influence of heterogeneous imaging parameters on the resulting radiomics signature, which ensures that any variability in the developed model is reflective of the underlying physiology/biology of the imaged lesion instead of being biased by inconsistent scanner protocols. ComBat is a commonly used harmonisation technique that employs an empirical Bayes framework to alter datasets to account for so-called ‘batch effects’, which refer to confounding experimental factors that affect the output of data other than the underlying biological variations that are of clinical interest (141). The method was originally introduced to reduce the influence of batch effects in genomic microarray research, but its applicability is not limited to this field and has since been utilised in radiomics research. Imaging acquisition protocols and reconstruction methods are batch effects in radiomics research that can be minimised by utilising the ComBat method, and evidence exists supporting its usefulness as a harmonisation technique for imaging modalities such as PET (142), CT (60, 143, 144), and MRI (145).

While the current evidence points to the usefulness of the ComBat harmonisation method, phantom studies confirming that the method improves feature reproducibility across scanner parameters in specific use cases should be performed where feasible. Indeed, it should be noted that there is no guarantee that the use of ComBat harmonisation, even if the method increases feature reproducibility across variable scanner parameters, will lead to a model with increased diagnostic, prognostic, or predictive power. This is demonstrated in a study by Peeken et al. (116) where the use of ComBat harmonisation resulted in an insignificant AUC value change of 0.01 in logistic regression models used to determine the malignancy status of segmented lymph nodes. Thus, while ComBat harmonisation can have a positive effect on the reproducibility of radiomics features, whether this leads to an improved model will depend on the particular modelling task and should be the subject of future research. Current mPCa radiomics research has experimented minimally with this technique, and future radiomics studies relating to mPCa should explore this technique further.

Studies reviewed in the present work utilised a myriad of different radiomics software for feature extraction. This is problematic for reproducibility, since it is known that even when extracting the same imaging biomarker, different software can yield different results (146). The standardisation of quantitative imaging biomarker definitions is therefore important to ensure maximum reproducibility. Standardisation initiatives such as the image biomarker standardisation initiative (IBSI) have attempted to address the problem of variable biomarker definitions, producing a set of standardised definitions that researchers can use for their quantitative imaging tasks (54, 147). Open source radiomics feature extraction software such as PyRadiomics and RaCaT (148, 149), implemented in the popular coding languages Python and C++, define biomarkers largely in accordance with the IBSI guidelines and carefully document any deviations, which can improve study reproducibility. Not specifying the software used or utilising in-house software that is not provided open source (and is therefore impossible to verify) should be minimised in favour of open-source projects such as those above in future works.

### 6.3 Deep Learning – The Future of Radiomics in mPCa?

The reliance on traditional hand-crafted features to characterise ROIs is a limiting factor in radiomics research that can be supplemented by using deep learning methods. Automated feature generation with saliency to the predictive task at hand is a notable benefit that can streamline the radiomics workflow, reducing the need for manual or data-driven feature selection techniques and enable end-to-end predictive modelling with limited human intervention required. The deep radiomics papers reviewed in this work demonstrate the ability of these algorithms to achieve good results in classifying the malignancy of overall patient scans (127, 139), classifying individual bone lesions (126), or detecting the anatomical locations of metastatic lesions throughout the patient body (99). As already discussed, external validation is overwhelmingly lacking and needs to be addressed, however, the literature available to date suggest the significant potential for deep learning to contribute positively to the clinical management of mPCa. It should be noted, however, that the overwhelming majority of deep radiomics papers relating to mPCa developed models with diagnostic applicability. Only a single study attempted to perform any analysis that had prognostic value (136), where they associated the number of automatically detected lymph nodes with PCa-specific survival (HR = 1.19, 95% CI 1.05 – 1.33). The heterogeneity of mPCa and plethora of available treatment options such as hormone therapy, chemotherapy, PSMA-labelled isotope therapy and others make this disease a prime target for deep modelling capable of predicting optimal treatment regimens (150). This remains a relatively unexplored pathway, and future work should certainly delve into this rich area.

Deep learning does not need to be utilised for end-to-end feature extraction and modelling to aid substantively with specific aspects of the radiomics workflow. ROI segmentation is one area where deep learning has already made significant contributions. The recent explosion of fully convolutional networks (FCNs) has led to the development of numerous algorithms capable of performing fully automated segmentation of anatomical structures and patient lesions. FCNs such as the U-Net and its variants have revolutionised the field of medical image segmentation through their ability to output state-of-the-art delineations in seconds (151–153). Kostyszyn et al. (154), for example, have demonstrated the possibility of fully automated prostatic gross tumour volume segmentation on PSMA-PET images in patients with primary PCa. They found good concordance between the fully automated segmentation and the expert manual contour on an external validation cohort, achieving a median dice similarity coefficient (DSC) of 0.81 (range: 0.32-0.95). Other studies have demonstrated the possibility of fully automated and accurate segmentation of the prostate gland and its associated zones with deep learning across various imaging modalities such as CT (155–157) and MRI (158–160). Zhao et al. (99) developed a modified 2.5D U-Net architecture for the automated segmentation of metastatic prostate lesions in ^68^Ga-PSMA PET/CT images in a proof-of-concept study. Their ensemble model, which leveraged information extracted from the axial, sagittal and coronal imaging planes simultaneously, was able to achieve mean DSCs of 0.645 and 0.544 for bone lesions and lymph node lesions when compared to expert manual segmentations, respectively, although the analysis was restricted to metastatic lesions contained in the pelvic area and fully-body validation has yet to be undertaken. By utilising fully automated segmentation algorithms reproducible segmentations can be produced and the issue of inter- and intra-observer variability is resolved, however, further research in this space is necessary because the lack of generalisability of these algorithms to independent datasets remains an impediment to their widespread clinical adoption (87).

Deep learning techniques can yield great benefits, but they are not without their downsides. In all cases the interpretability of the model is compromised relative to the traditional radiomics pathway. While hand-crafted features can often relate to very specific and clinically understandable aspects of tumour biology, deep features are highly abstract and difficult for humans to interpret. A generalised statement on whether this is an acceptable downside cannot be made, as it depends primarily on what the desired outcome of the clinical model is, and whether clinicians value having a greater intuitive understanding of the results of a particular model. Also, the complexity of deep learning models demands greater amounts of training data to produce acceptable results without overfitting. Techniques exist to mitigate against overfitting, such as dropout regularization (161), batch normalization (162) and artificially increasing the size of the dataset through data augmentation. Nevertheless it remains true that the vast majority of current models do not demonstrate generalisability to external datasets, which is evidenced by the studies reviewed in the present work. These downsides need to be considered when future radiomics studies in mPCa are undertaken.

### 6.4 Hybrid Imaging

There is no inherent limitation on the number of imaging modalities from which radiomics features can be extracted for use in predictive modelling. Hybrid imaging techniques capture expanded amounts of information that can be complementary in nature, where each modality characterises different information about the underlying biology of the tumour (163). ^68^Ga-PSMA PET/CT imaging, for example, plays a crucial role in the detection and subsequent clinical management of metastatic PCa (11). ^68^Ga-PSMA PET captures physiological information within the patient’s body about the distribution of the PSMA receptor, which is substantially overexpressed in the vast majority of PCa cases (17), while the CT provides high-resolution imaging reflecting the underlying density of the patient anatomy. Radiomics features extracted from each of these modalities will thus characterise the heterogeneity of the tumour in different and potentially complementary ways, which could improve model performance. There is evidence that utilising this approach in mPCa radiomics modelling can yield good results, both in the traditional radiomics methodology (93, 95, 98) and deep radiomics (99). Particularly in the field of deep radiomics, where the analysis of dual modalities can often be as simple as incorporating an additional channel in the network architecture, this method of analysis should be thoroughly explored.

## Conclusion

Radiomics analysis, both using hand-crafted and machine-crafted features has demonstrated significant diagnostic, prognostic, and predictive potential in the clinical management of mPCa. Quality assessment of the identified studies, however, revealed major limitations preventing the implementation of these models in routine clinical practice. Future work should conduct multi-centre and prospective validation of developed radiomics models as a priority to facilitate the clinical translation of radiomics models, so that the full potential of this field can be realised.

## Author Contributions

Conception of study: JK, PR, GH, RF, and ME. Collecting literature: JK. Writing Manuscript: JK. Reviewing manuscript: JK, RF, GH, PR, RJ, CK, BR, and ME. All authors contributed to the article and approved the submitted version.

## Funding

The authors would like to acknowledge the funding support from the Royal Perth Imaging Research PhD Fellowship (Grant Number 0010121).

## Conflict of Interest

The authors declare that the research was conducted in the absence of any commercial or financial relationships that could be construed as a potential conflict of interest.

## Publisher’s Note

All claims expressed in this article are solely those of the authors and do not necessarily represent those of their affiliated organizations, or those of the publisher, the editors and the reviewers. Any product that may be evaluated in this article, or claim that may be made by its manufacturer, is not guaranteed or endorsed by the publisher.

## Footnotes

1
https://github.com/

2
https://www.mskcc.org/nomograms/prostate

3 Keras: https://keras.io/

4 Tensorflow: https://www.tensorflow.org/

5 PyTorch: https://pytorch.org/

## References

[1] Sung H Ferlay J Siegel RL Laversanne M Soerjomataram I Jemal A. Global Cancer Statistics 2020: GLOBOCAN Estimates of Incidence and Mortality Worldwide for 36 Cancers in 185 Countries. CA Cancer J Clin (2021) 71(3):209–49. doi: 10.3322/caac.21660 33538338

[2] Norum J Nieder C. Treatments for Metastatic Prostate Cancer (mPC): A Review of Costing Evidence. Pharmacoeconomics (2017) 35(12):1223–36. doi: 10.1007/s40273-017-0555-8 28756597

[3] Chaffer CL Weinberg RA. A Perspective on Cancer Cell Metastasis. Science (2011) 331(6024):1559–64. doi: 10.1126/science.1203543 21436443

[4] Svensson E Christiansen CF Ulrichsen SP Rørth MR Sørensen HT. Survival After Bone Metastasis by Primary Cancer Type: A Danish Population-Based Cohort Study. BMJ Open (2017) 7(9):e016022. doi: 10.1136/bmjopen-2017-016022 PMC5595184 28893744

[5] Daneshmand S Quek ML Stein JP Lieskovsky G Cai JIE Pinski J. Prognosis of Patients With Lymph Node Positive Prostate Cancer Following Radical Prostatectomy: Long Term Results. J Urol (2004) 172(6):2252–5. doi: 10.1097/01.ju.0000143448.04161.cc 15538242

[6] Yadav SS Stockert JA Hackert V Yadav KK Tewari AK. Intratumor Heterogeneity in Prostate Cancer. Urol Oncol (2018) 36(8):349–60. doi: 10.1016/j.urolonc.2018.05.008 29887240

[7] Ghafoor S Burger IA Vargas AH. Multimodality Imaging of Prostate Cancer. J Nucl Med (2019) 60(10):1350–8. doi: 10.2967/jnumed.119.228320 PMC6785785 31481573

[8] Tourinho-Barbosa RR Srougi V Nunes-Silva I Baghdadi M Rembeyo G Eiffel SS. Biochemical Recurrence After Radical Prostatectomy: What Does it Mean? Int Braz J Urol (2018) 44(1):14–21. doi: 10.1590/S1677-5538.IBJU.2016.0656 29039897 PMC5815528

[9] Mottet N Bellmunt J Bolla M Briers E Cumberbatch MG De Santis M. EAU-ESTRO-SIOG Guidelines on Prostate Cancer. Part 1: Screening, Diagnosis, and Local Treatment With Curative Intent. Eur Urol (2016) 71(4):618–29. doi: 10.1016/j.eururo.2016.08.003 27568654

[10] Cui T Kovell RC Terlecki RP. Is it Time to Abandon the Digital Rectal Examination? Lessons From the PLCO Cancer Screening Trial and Peer-Reviewed Literature. Curr Med Res Opin (2016) 32(10):1663–9. doi: 10.1080/03007995.2016.1198312 27264113

[11] Lenzo NP Meyrick D Turner JH. Review of Gallium-68 PSMA PET/CT Imaging in the Management of Prostate Cancer. Diagnostics (Basel) (2018) 8(1):16. doi: 10.3390/diagnostics8010016 PMC5871999 29439481

[12] Thompson IM Pauler DK Goodman PJ Tangen CM Lucia MS Parnes HL. Prevalence of Prostate Cancer Among Men With a Prostate-Specific Antigen Level ≤4.0 Ng Per Milliliter. N Engl J Med (2004) 350(22):2239–46. doi: 10.1056/NEJMoa031918 15163773

[13] Epstein JI Feng Z Trock BJ Pierorazio PM. Upgrading and Downgrading of Prostate Cancer From Biopsy to Radical Prostatectomy: Incidence and Predictive Factors Using the Modified Gleason Grading System and Factoring in Tertiary Grades. Eur Urol (2012) 61(5):1019–24. doi: 10.1016/j.eururo.2012.01.050 PMC4659370 22336380

[14] Van den Wyngaert T Strobel K Kampen WU Kuwert T van der Bruggen W Mohan HK. The EANM Practice Guidelines for Bone Scintigraphy. Eur J Nucl Med Mol Imaging (2016) 43(9):1723–38. doi: 10.1007/s00259-016-3415-4 PMC4932135 27262701

[15] Pedler K Kitzing YX Varol C Arianayagam M. The Current Status of MRI in Prostate Cancer. Aust Fam Physician (2015) 44(4):225–30. 25901408

[16] Ahmed HUF El-Shater Bosaily AM Brown LCP Gabe RP Kaplan RP Parmar MKP. Diagnostic Accuracy of Multi-Parametric MRI and TRUS Biopsy in Prostate Cancer (PROMIS): A Paired Validating Confirmatory Study. Lancet (2017) 389(10071):815–22. doi: 10.1016/S0140-6736(16)32401-1 28110982

[17] Rajasekaran SA Anilkumar G Oshima E Bowie JU Liu H Heston W. A Novel Cytoplasmic Tail MXXXL Motif Mediates the Internalization of Prostate-Specific Membrane Antigen. Mol Biol Cell (2003) 14(12):4835–45. doi: 10.1091/mbc.E02-11-0731 PMC284788 14528023

[18] Afshar-Oromieh A Avtzi E Giesel FL Holland-Letz T Linhart HG Eder M. The Diagnostic Value of PET/CT Imaging With the 68Ga-Labelled PSMA Ligand HBED-CC in the Diagnosis of Recurrent Prostate Cancer. Eur J Nucl Med Mol Imaging (2015) 42(2):197–209. doi: 10.1007/s00259-014-2949-6 25411132 PMC4315487

[19] Morigi JJ Stricker PD Van Leeuwen PJ Tang R Ho B Nguyen Q. Prospective Comparison of 18F-Fluoromethylcholine Versus 68ga-PSMA PET/CT in Prostate Cancer Patients Who Have Rising PSA After Curative Treatment and are Being Considered for Targeted Therapy. J Nucl Med (2015) 56(8):1185–90. doi: 10.2967/jnumed.115.160382 26112024

[20] Kumar V Gu Y Basu S Berglund A Eschrich SA Schabath MB. Radiomics: The Process and the Challenges. Magn Reson Imaging (2012) 30(9):1234–48. doi: 10.1016/j.mri.2012.06.010 PMC3563280 22898692

[21] Gillies RJ Kinahan PE Hricak H. Radiomics: Images Are More Than Pictures, They Are Data. Radiology (2016) 278(2):563–77. doi: 10.1148/radiol.2015151169 PMC4734157 26579733

[22] Yip SSF Aerts HJWL. Applications and Limitations of Radiomics. Phys Med Biol (2016) 61(13):R150–R66. doi: 10.1088/0031-9155/61/13/R150 PMC4927328 27269645

[23] Zhao B Lee SM Lee H-J Tan Y Qi J Persigehl T. Variability in Assessing Treatment Response: Metastatic Colorectal Cancer as a Paradigm. Clin Cancer Res (2014) 20(13):3560–8. doi: 10.1158/1078-0432.CCR-14-0245 PMC4337392 24780294

[24] Mazor RD Savir A Gheorghiu D Weinstein Y Abadi-Korek I Shabshin N. The Inter-Observer Variability of Breast Density Scoring Between Mammography Technologists and Breast Radiologists and its Effect on the Rate of Adjuvant Ultrasound. Eur J Radiol (2016) 85(5):957–62. doi: 10.1016/j.ejrad.2016.02.023 27130056

[25] Djavan BOB Waldert M Zlotta A Dobronski P Seitz C Remzi M. Safety and Morbidity of First and Repeat Transrectal Ultrasound Guided Prostate Needle Biopsies: Results of a Prospective European Prostate Cancer Detection Study. J Urol (2001) 166(3):856–60. doi: 10.1016/S0022-5347(05)65851-X 11490233

[26] Lambin P Rios-Velazquez E Leijenaar R Carvalho S van Stiphout RGPM Granton P. Radiomics: Extracting More Information From Medical Images Using Advanced Feature Analysis. Eur J Cancer (1990) (2011) 48(4):441–6. doi: 10.1016/j.ejca.2011.11.036 PMC4533986 22257792

[27] Asselin M-C O’Connor JPB Boellaard R Thacker NA Jackson A. Quantifying Heterogeneity in Human Tumours Using MRI and PET. Eur J Cancer (2012) 48(4):447–55. doi: 10.1016/j.ejca.2011.12.025 22265426

[28] Sun Y Reynolds HM Parameswaran B Wraith D Finnegan ME Williams S. Multiparametric MRI and Radiomics in Prostate Cancer: A Review. Australas Phys Eng Sci Med (2019) 42(1):3–25. doi: 10.1007/s13246-019-00730-z 30762223

[29] Xue C Zhou Y Lo GG Wong OL Yu SK Cheung KY. Reliability of Radiomics Features Due to Image Reconstruction Using a Standardized T2-Weighted Pulse Sequence for MR-Guided Radiotherapy: An Anthropomorphic Phantom Study. Magn Reson Med (2021) 85(6):3434–46. doi: 10.1002/mrm.28650 33404129

[30] Shafiq-Ul-Hassan M Latifi K Zhang G Ullah G Gillies R Moros E. Voxel Size and Gray Level Normalization of CT Radiomic Features in Lung Cancer. Sci Rep (2018) 8(1):10545–9. doi: 10.1038/s41598-018-28895-9 PMC6043486 30002441

[31] Zhao B. Understanding Sources of Variation to Improve the Reproducibility of Radiomics. Front Oncol (2021) 11:633176. doi: 10.3389/fonc.2021.633176 33854969 PMC8039446

[32] Lambin P Leijenaar RTH Deist TM Peerlings J de Jong EEC van Timmeren J. Radiomics: The Bridge Between Medical Imaging and Personalized Medicine. Nat Rev Clin Oncol (2017) 14(12):749–62. doi: 10.1038/nrclinonc.2017.141 28975929

[33] Collins GS Reitsma JB Altman DG Moons KGM. Transparent Reporting of a Multivariable Prediction Model for Individual Prognosis or Diagnosis (TRIPOD) : The TRIPOD Statement. BMJ (2015) 350:g7594. doi: 10.1136/bmj.g7594 25569120

[34] Park JE Kim D Kim HS Park SY Kim JY Cho SJ. Quality of Science and Reporting of Radiomics in Oncologic Studies: Room for Improvement According to Radiomics Quality Score and TRIPOD Statement. Eur Radiol (2020) 30(1):523–36. doi: 10.1007/s00330-019-06360-z 31350588

[35] Fiz F Viganò L Gennaro N Costa G La Bella L Boichuk A. Radiomics of Liver Metastases: A Systematic Review. Cancers (Basel) (2020) 12(10):1–24. doi: 10.3390/cancers12102881 PMC7600822 33036490

[36] Stanzione A Gambardella M Cuocolo R Ponsiglione A Romeo V Imbriaco M. Prostate MRI Radiomics: A Systematic Review and Radiomic Quality Score Assessment. Eur J Radiol (2020) 129:109095. doi: 10.1016/j.ejrad.2020.109095 32531722

[37] Park JE Kim HS Kim D Park SY Kim JY Cho SJ. A Systematic Review Reporting Quality of Radiomics Research in Neuro-Oncology: Toward Clinical Utility and Quality Improvement Using High-Dimensional Imaging Features. BMC Cancer (2020) 20(1):29. doi: 10.1186/s12885-019-6504-5 31924170 PMC6954557

[38] Chetan MR Gleeson FV. Radiomics in Predicting Treatment Response in non-Small-Cell Lung Cancer: Current Status, Challenges and Future Perspectives. Eur Radiol (2021) 31(2):1049–58. doi: 10.1007/s00330-020-07141-9 PMC7813733 32809167

[39] Zhao B Tan Y Tsai W-Y Qi J Xie C Lu L. Reproducibility of Radiomics for Deciphering Tumor Phenotype With Imaging. Sci Rep (2016) 6(1):23428. doi: 10.1038/srep23428 27009765 PMC4806325

[40] Shafiq-ul-Hassan M Zhang GG Latifi K Ullah G Hunt DC Balagurunathan Y. Intrinsic Dependencies of CT Radiomic Features on Voxel Size and Number of Gray Levels. Med Phys (2017) 44(3):1050–62. doi: 10.1002/mp.12123 PMC5462462 28112418

[41] Zwanenburg A. Radiomics in Nuclear Medicine: Robustness, Reproducibility, Standardization, and How to Avoid Data Analysis Traps and Replication Crisis. Eur J Nucl Med Mol Imaging (2019) 46(13):2638–55. doi: 10.1007/s00259-019-04391-8 31240330

[42] Midya A Chakraborty J Gönen M Do RKG Simpson AL. Influence of CT Acquisition and Reconstruction Parameters on Radiomic Feature Reproducibility. J Med Imaging (2018) 5(1):11020. doi: 10.1117/1.JMI.5.1.011020 PMC5812985 29487877

[43] Berenguer R Del Rosario Pastor-Juan M Canales-Vázquez J Castro-García M Villas MV Legorburo FM. Radiomics of CT Features may be Nonreproducible and Redundant: Influence of CT Acquisition Parameters. Radiology (2018) 288(2):407–15. doi: 10.1148/radiol.2018172361 29688159

[44] Li X Chen E Guo B Yang W Han R Hu C. The Impact of Respiratory Motion and CT Pitch on the Robustness of Radiomics Feature Extraction in 4DCT Lung Imaging. Comput Methods Programs BioMed (2020) 197:105719. doi: 10.1016/j.cmpb.2020.105719 32916542

[45] Larue RTHM van Timmeren JE de Jong EEC Feliciani G Leijenaar RTH Schreurs WMJ. Influence of Gray Level Discretization on Radiomic Feature Stability for Different CT Scanners, Tube Currents and Slice Thicknesses: A Comprehensive Phantom Study. Acta Oncol (2017) 56(11):1544–53. doi: 10.1080/0284186X.2017.1351624 28885084

[46] Mahmood U Apte A Deasy JO Schmidtlein CR Shukla-Dave A. Investigating the Robustness Neighborhood Grey Tone Difference Matrix (NGTDM) and Grey Level Co-Occurrence Matrix (GLCM) Radiomic Features on Clinical Computed Tomography Systems Using Anthropomorphic Phantoms: Evidence From a Multivendor Study. J Comput Assist Tomogr (2017) 41(6):995. doi: 10.1097/RCT.0000000000000632 28708732 PMC5685887

[47] Park JE Park SY Kim HJ Kim HS. Reproducibility and Generalizability in Radiomics Modeling: Possible Strategies in Radiologic and Statistical Perspectives. Korean J Radiol (2019) 20(7):1124–37. doi: 10.3348/kjr.2018.0070 PMC6609433 31270976

[48] Shur J Blackledge M D’Arcy J Collins DJ Bali M O’Leach M. MRI Texture Feature Repeatability and Image Acquisition Factor Robustness, a Phantom Study and in Silico Study. Eur Radiol Exp (2021) 5(1):2. doi: 10.1186/s41747-020-00199-6 33462642 PMC7813908

[49] Yang F Dogan N Stoyanova R Ford JC. Evaluation of Radiomic Texture Feature Error Due to MRI Acquisition and Reconstruction: A Simulation Study Utilizing Ground Truth. Phys Med (2018) 50:26–36. doi: 10.1016/j.ejmp.2018.05.017 29891091

[50] Molina D Pérez-Beteta J Martínez-González A Martino J Velasquez C Arana E. Influence of Gray Level and Space Discretization on Brain Tumor Heterogeneity Measures Obtained From Magnetic Resonance Images. Comput Biol Med (2016) 78:49–57. doi: 10.1016/j.compbiomed.2016.09.011 27658261

[51] Montagne S Hamzaoui D Allera A Ezziane M Luzurier A Quint R. Challenge of Prostate MRI Segmentation on T2-Weighted Images: Inter-Observer Variability and Impact of Prostate Morphology. Insights Imaging (2021) 12(1):71. doi: 10.1186/s13244-021-01010-9 PMC8179870 34089410

[52] Fiorino C Reni M Bolognesi A Cattaneo GM Calandrino R. Intra- and Inter-Observer Variability in Contouring Prostate and Seminal Vesicles: Implications for Conformal Treatment Planning. Radiother Oncol (1998) 47(3):285–92. doi: 10.1016/S0167-8140(98)00021-8 9681892

[53] Balagurunathan Y Gu Y Wang H Kumar V Grove O Hawkins S. Reproducibility and Prognosis of Quantitative Features Extracted From CT Images. Transl Oncol (2014) 7(1):72–87. doi: 10.1593/tlo.13844 24772210 PMC3998690

[54] Zwanenburg A Leger S Vallières M Löck S. Image Biomarker Standardisation Initiative. arXiv:161207003 (2016).

[55] Leijenaar RTH Nalbantov G Carvalho S van Elmpt WJC Troost EGC Boellaard R. The Effect of SUV Discretization in Quantitative FDG-PET Radiomics: The Need for Standardized Methodology in Tumor Texture Analysis. Sci Rep (2015) 5(1):11075. doi: 10.1038/srep11075 26242464 PMC4525145

[56] Altazi BA Zhang GG Fernandez DC Montejo ME Hunt D Werner J. Reproducibility of F18-FDG PET Radiomic Features for Different Cervical Tumor Segmentation Methods, Gray-Level Discretization, and Reconstruction Algorithms. J Appl Clin Med Phys (2017) 18(6):32–48. doi: 10.1002/acm2.12170 PMC5689938 28891217

[57] Hatt M Tixier F Pierce L Kinahan PE Le Rest CC Visvikis D. Characterization of PET/CT Images Using Texture Analysis: The Past, the Present… Any Future? Eur J Nucl Med Mol Imaging (2017) 44(1):151–65. doi: 10.1007/s00259-016-3427-0 PMC5283691 27271051

[58] van Velden FHP Kramer GM Frings V Nissen IA Mulder ER de Langen AJ. Repeatability of Radiomic Features in Non-Small-Cell Lung Cancer [F-18]FDG-PET/CT Studies: Impact of Reconstruction and Delineation. Mol Imaging Biol (2016) 18(5):788–95. doi: 10.1007/s11307-016-0940-2 PMC5010602 26920355

[59] Duron L Balvay D Perre SV Bouchouicha A Savatovsky J Sadik J-C. Gray-Level Discretization Impacts Reproducible MRI Radiomics Texture Features. PloS One (2019) 14(3):e0213459. doi: 10.1371/journal.pone.0213459 30845221 PMC6405136

[60] Ibrahim A Refaee T Primakov S Barufaldi B Acciavatti RJ Granzier RWY. The Effects of In-Plane Spatial Resolution on CT-Based Radiomic Features' Stability With and Without ComBat Harmonization. Cancers (Basel) (2021) 13(8):1848. doi: 10.3390/cancers13081848 33924382 PMC8103509

[61] Whybra P Parkinson C Foley K Staffurth J Spezi E. Assessing Radiomic Feature Robustness to Interpolation in 18F-FDG PET Imaging. Sci Rep (2019) 9(1):1–10. doi: 10.1038/s41598-019-46030-0 31273242 PMC6609613

[62] Park S-H Lim H Bae BK Hahm MH Chong GO Jeong SY. Robustness of Magnetic Resonance Radiomic Features to Pixel Size Resampling and Interpolation in Patients With Cervical Cancer. Cancer Imaging (2021) 21(1):19. doi: 10.1186/s40644-021-00388-5 33531073 PMC7856733

[63] Avanzo M Wei L Stancanello J Vallieres M Rao A Morin O. Machine and Deep Learning Methods for Radiomics. Med Phys (2020) 47(5):e185–202. doi: 10.1002/mp.13678 PMC8965689 32418336

[64] LeCun Y Bengio Y Hinton G. Deep Learning. Nature (2015) 521(7553):436–44. doi: 10.1038/nature14539 26017442

[65] Naqa IE. The Role of Quantitative PET in Predicting Cancer Treatment Outcomes. Clin Transl Imaging (2014) 2(4):305–20. doi: 10.1007/s40336-014-0063-1

[66] Wei L El Naqa I. Feature Extraction and Qualification. In: Radiomics and Radiogenomics. Boca Raton: Chapman and Hall/CRC (2019). p. 121–49.

[67] Jain AK. Fundamentals of Digital Image Processing. Englewood Cliffs:Prentice-Hall (1989).

[68] Haralick RM Shanmugam K Dinstein IH. Textural Features for Image Classification. IEEE Trans Syst Man Cybern (1973) 6):610–21. doi: 10.1109/TSMC.1973.4309314

[69] Galloway MM. Texture Analysis Using Gray Level Run Lengths. Comput Gr Image Process (1975) 4(2):172–9. doi: 10.1016/S0146-664X(75)80008-6

[70] Thibault G Angulo J Meyer F. Advanced Statistical Matrices for Texture Characterization: Application to Cell Classification. IEEE Trans BioMed Eng (2014) 61(3):630–7. doi: 10.1109/TBME.2013.2284600 24108747

[71] Amadasun M King R. Textural Features Corresponding to Textural Properties. IEEE Trans Syst Man Cybern (1989) 19(5):1264–74. doi: 10.1109/21.44046

[72] Sun C Wee WG. Neighboring Gray Level Dependence Matrix for Texture Classification. Comput Gr Image Process (1983) 23(3):341–52. doi: 10.1016/0734-189X(83)90032-4

[73] Rizzo S Botta F Raimondi S Origgi D Fanciullo C Morganti AG. Radiomics: The Facts and the Challenges of Image Analysis. Eur Radiol Exp (2018) 2(1):1–8. doi: 10.1186/s41747-018-0068-z 30426318 PMC6234198

[74] Fogel I Sagi D. Gabor Filters as Texture Discriminator. Biol Cybern (1989) 61(2):103–13. doi: 10.1007/BF00204594

[75] Addison PS. The Illustrated Wavelet Transform Handbook. In: Raton B, editor. Introductory Theory and Applications in Science, Engineering, Medicine and Finance, 2nd ed. Boca Raton: Taylor & Francis Group (2016).

[76] Noortman WA Vriens D Slump CH Bussink J Meijer TW de Geus-Oei L-F. Adding the Temporal Domain to PET Radiomic Features. PloS One (2020) 15(9):e0239438. doi: 10.1371/journal.pone.0239438 32966313 PMC7510999

[77] Goodfellow I Bengio Y Courville A. Deep Learning. Cambridge: MIT press (2016).

[78] Zeiler MD Fergus R eds. Visualizing and Understanding Convolutional Networks. In: European Conference on Computer Vision. Zurich: Springer.

[79] Shang S Sun J Yue Z Wang Y Wang X Luo Y. Multi-Parametric MRI Based Radiomics With Tumor Subregion Partitioning for Differentiating Benign and Malignant Soft-Tissue Tumors. BioMed Signal Process Control (2021) 67:102522. doi: 10.1016/j.bspc.2021.102522

[80] Lao J Chen Y Li Z-C Li Q Zhang J Liu J. A Deep Learning-Based Radiomics Model for Prediction of Survival in Glioblastoma Multiforme. Sci Rep (2017) 7(1):10353–8. doi: 10.1038/s41598-017-10649-8 PMC5583361 28871110

[81] Avanzo M Stancanello J Pirrone G Sartor G. Radiomics and Deep Learning in Lung Cancer. Strahlenther Onkol (2020) 196(10):879–87. doi: 10.1007/s00066-020-01625-9 32367456

[82] Hou Y Bao J Song Y Bao M-L Jiang K-W Zhang J. Integration of Clinicopathologic Identification and Deep Transferrable Image Feature Representation Improves Predictions of Lymph Node Metastasis in Prostate Cancer. EBioMedicine (2021) 68:103395. doi: 10.1016/j.ebiom.2021.103395 34049247 PMC8167242

[83] Stevens LM Mortazavi BJ Deo RC Curtis L Kao DP. Recommendations for Reporting Machine Learning Analyses in Clinical Research. Circ Cardiovasc Qual Outcomes (2020) 13(10):e006556. doi: 10.1161/CIRCOUTCOMES.120.006556 33079589 PMC8320533

[84] Yao S Jiang H Song B. Radiomics in Prostate Cancer: Basic Concepts and Current State-of-the-Art. Chin J Acad Radiol (2020) 2(3):47–55. doi: 10.1007/s42058-019-00020-3

[85] Sullivan DC Obuchowski NA Kessler LG Raunig DL Gatsonis C Huang EP. Metrology Standards for Quantitative Imaging Biomarkers. Radiology (2015) 277(3):813–25. doi: 10.1148/radiol.2015142202 PMC4666097 26267831

[86] Osman SOS Leijenaar RTH Cole AJ Lyons CA Hounsell AR Prise KM. Computed Tomography-Based Radiomics for Risk Stratification in Prostate Cancer. Int J Radiat Oncol Biol Phys (2019) 105(2):448–56. doi: 10.1016/j.ijrobp.2019.06.2504 31254658

[87] van Timmeren JE Cester D Tanadini-Lang S Alkadhi H Baessler B. Radiomics in Medical Imaging—”How-to” Guide and Critical Reflection. Insights Imaging (2020) 11(1):91. doi: 10.1186/s13244-020-00887-2 32785796 PMC7423816

[88] Obuchowski NA Reeves AP Huang EP Wang X-F Buckler AJ Kim HJ. Quantitative Imaging Biomarkers: A Review of Statistical Methods for Computer Algorithm Comparisons. Stat Methods Med Res (2015) 24(1):68–106. doi: 10.1177/0962280214537390 24919829 PMC4263694

[89] Lin C Harmon S Bradshaw T Eickhoff J Perlman S Liu G. Response-To-Repeatability of Quantitative Imaging Features for Longitudinal Response Assessment. Phys Med Biol (2019) 64(2):025019. doi: 10.1088/1361-6560/aafa0a 30566922

[90] Tzeng S Zhu J Weisman AJ Bradshaw TJ Jeraj R. Spatial Process Decomposition for Quantitative Imaging Biomarkers Using Multiple Images of Varying Shapes. Stat Med (2021) 40(5):1243–61. doi: 10.1002/sim.8838 PMC8848296 33336451

[91] Liu X Khalvati F Namdar K Fischer S Lewis S Taouli B. Can Machine Learning Radiomics Provide Pre-Operative Differentiation of Combined Hepatocellular Cholangiocarcinoma From Hepatocellular Carcinoma and Cholangiocarcinoma to Inform Optimal Treatment Planning? Eur Radiol (2021) 31(1):244–55. doi: 10.1007/s00330-020-07119-7 32749585

[92] Zhang Y Oikonomou A Wong A Haider MA Khalvati F. Radiomics-Based Prognosis Analysis for non-Small Cell Lung Cancer. Sci Rep (2017) 7(1):1–8. doi: 10.1038/srep46349 28418006 PMC5394465

[93] Damascelli A Gallivanone F Cristel G Cava C Interlenghi M Esposito A. Advanced Imaging Analysis in Prostate MRI: Building a Radiomic Signature to Predict Tumor Aggressiveness. Diagnostics (2021) 11(4):594. doi: 10.3390/diagnostics11040594 33810222 PMC8065545

[94] Wang Y Yu B Zhong F Guo Q Li K Hou Y. MRI-Based Texture Analysis of the Primary Tumor for Pre-Treatment Prediction of Bone Metastases in Prostate Cancer. Magn Reson Imaging (2019) 60:76–84. doi: 10.1016/j.mri.2019.03.007 30917943

[95] Zhang W Mao N Wang Y Xie H Duan S Zhang X. A Radiomics Nomogram for Predicting Bone Metastasis in Newly Diagnosed Prostate Cancer Patients. Eur J Radiol (2020) 128:109020. doi: 10.1016/j.ejrad.2020.109020 32371181

[96] Acar E Leblebici A Ellidokuz BE Basbinar Y Kaya GC. Machine Learning for Differentiating Metastatic and Completely Responded Sclerotic Bone Lesion in Prostate Cancer: A Retrospective Radiomics Study. Br J Radiol (2019) 92(1101):20190286. doi: 10.1259/bjr.20190286 31219712 PMC6732932

[97] Cysouw MCF Jansen BHE van de Brug T Oprea-Lager DE Pfaehler E de Vries BM. Machine Learning-Based Analysis of [18F]DCFPyL PET Radiomics for Risk Stratification in Primary Prostate Cancer. Eur J Nucl Med Mol Imaging (2021) 48(2):340–9. doi: 10.1007/s00259-020-04971-z PMC7835295 32737518

[98] Perk T Bradshaw T Chen S Im H-J Cho S Perlman S. Automated Classification of Benign and Malignant Lesions in 18 F-NaF PET/CT Images Using Machine Learning. Phys Med Biol (2018) 63(22):225019. doi: 10.1088/1361-6560/aaebd0 30457118

[99] Zhao Y Gafita A Vollnberg B Tetteh G Haupt F Afshar-Oromieh A. Deep Neural Network for Automatic Characterization of Lesions on 68Ga-PSMA-11 PET/CT. Eur J Nucl Med Mol Imaging (2020) 47(3):603–13. doi: 10.1007/s00259-019-04606-y 31813050

[100] Cheng D-C Hsieh T-C Yen K-Y Kao C-H. Lesion-Based Bone Metastasis Detection in Chest Bone Scintigraphy Images of Prostate Cancer Patients Using Pre-Train, Negative Mining, and Deep Learning. Diagnostics (2021) 11(3):518. doi: 10.3390/diagnostics11030518 33803921 PMC8000593

[101] Horwich A Parker C de Reijke T Kataja V. Prostate Cancer: ESMO Clinical Practice Guidelines for Diagnosis, Treatment and Follow-Up. Ann Oncol (2013) 24(Suppl. 6):106–14. doi: 10.1093/annonc/mdt208 23813930

[102] Vickers AJ Ulmert D Sjoberg DD Bennette CJ Björk T Gerdtsson A. Strategy for Detection of Prostate Cancer Based on Relation Between Prostate Specific Antigen at Age 40-55 and Long Term Risk of Metastasis: Case-Control Study. BMJ (2013) 346(7907):27–f2023. doi: 10.1136/bmj.f2023 PMC3933251 23596126

[103] Khurshid Z Ahmadzadehfar H Gaertner FC Papp L Zsóter N Essler M. Role of Textural Heterogeneity Parameters in Patient Selection for 177Lu-PSMA Therapy. via response prediction Oncotarget (2018) 9(70):33312–21. doi: 10.18632/oncotarget.26051 PMC6161784 30279962

[104] Rusthoven CGMD Carlson JAMD Waxweiler TVMD Yeh NMDPD Raben DMD Flaig TWMD. The Prognostic Significance of Gleason Scores in Metastatic Prostate Cancer. Urol Oncol (2014) 32(5):707–13. doi: 10.1016/j.urolonc.2014.01.004 24629494

[105] Loblaw DA Virgo KS Nam R Somerfield MR Ben-Josef E Mendelson DS. Initial Hormonal Management of Androgen-Sensitive Metastatic, Recurrent, or Progressive Prostate Cancer: 2006 Update of an American Society of Clinical Oncology Practice Guideline. J Clin Oncol (2007) 25(12):1596–605. doi: 10.1200/JCO.2006.10.1949 17404365

[106] Halabi S Small EJ Kantoff PW Kattan MW Kaplan EB Dawson NA. Prognostic Model for Predicting Survival in Men With Hormone-Refractory Metastatic Prostate Cancer. J Clin Oncol (2003) 21(7):1232–7. doi: 10.1200/JCO.2003.06.100 12663709

[107] Armstrong AJ Halabi S Luo J Nanus DM Giannakakou P Szmulewitz RZ. Prospective Multicenter Validation of Androgen Receptor Splice Variant 7 and Hormone Therapy Resistance in High-Risk Castration-Resistant Prostate Cancer: The PROPHECY Study. J Clin Oncol (2019) 37(13):1120–9. doi: 10.1200/JCO.18.01731 PMC6494355 30865549

[108] Oyama N Akino H Suzuki Y Kanamaru H Miwa Y Tsuka H. Prognostic Value of 2-Deoxy-2-[F-18] Fluoro-D-Glucose Positron Emission Tomography Imaging for Patients With Prostate Cancer. Mol Imaging Biol (2002) 4(1):99–104. doi: 10.1016/s1095-0397(01)00065-6 14538053

[109] Meirelles GSP SchÖDer H Ravizzini GC GÖNen M Fox JJ Humm J. Prognostic Value of Baseline [18f] Fluorodeoxyglucose Positron Emission Tomography and 99mtc-MDP Bone Scan in Progressing Metastatic Prostate Cancer. Clin Cancer Res (2010) 16(24):6093–9. doi: 10.1158/1078-0432.CCR-10-1357 PMC3402086 20975102

[110] Bauckneht M Bertagna F Donegani MI Durmo R Miceli A De Biasi V. The Prognostic Power of 18F-FDG PET/CT Extends to Estimating Systemic Treatment Response Duration in Metastatic Castration-Resistant Prostate Cancer (mCRPC) Patients. Prostate Cancer Prostatic Dis (2021). doi: 10.1038/s41391-021-00391-8 PMC8616756 34012060

[111] Moher D Liberati A Tetzlaff J Altman DG. Preferred Reporting Items for Systematic Reviews and Meta-Analyses: The PRISMA Statement. BMJ (2009) 339(7716):e78–336. doi: 10.1136/bmj.b2535 PMC3090117 21603045

[112] Alongi P Stefano A Comelli A Laudicella R Scalisi S Arnone G. Radiomics Analysis of 18F-Choline PET/CT in the Prediction of Disease Outcome in High-Risk Prostate Cancer: An Explorative Study on Machine Learning Feature Classification in 94 Patients. Eur Radiol (2021) 31(7):4595–605. doi: 10.1007/s00330-020-07617-8 33443602

[113] Hayakawa T Tabata K-i Tsumura H Kawakami S Katakura T Hashimoto M. Size of Pelvic Bone Metastasis as a Significant Prognostic Factor for Metastatic Prostate Cancer Patients. Japanese J Radiol (2020) 38(10):993–6. doi: 10.1007/s11604-020-01004-5 32537698

[114] Moazemi S Khurshid Z Erle A Lütje S Essler M Schultz T. Machine Learning Facilitates Hotspot Classification in PSMA-PET/CT With Nuclear Medicine Specialist Accuracy. Diagnostics (2020) 10(9):622. doi: 10.3390/diagnostics10090622 PMC7555620 32842599

[115] Moazemi S Erle A Lütje S Gaertner FC Essler M Bundschuh RA. Estimating the Potential of Radiomics Features and Radiomics Signature From Pretherapeutic PSMA-PET-CT Scans and Clinical Data for Prediction of Overall Survival When Treated With 177Lu-PSMA. Diagnostics (2021) 11(2):186. doi: 10.3390/diagnostics11020186 33525456 PMC7912143

[116] Peeken JC Shouman MA Kroenke M Rauscher I Maurer T Gschwend JE. A CT-Based Radiomics Model to Detect Prostate Cancer Lymph Node Metastases in PSMA Radioguided Surgery Patients. Eur J Nucl Med Mol Imaging (2020) 47(13):2968–77. doi: 10.1007/s00259-020-04864-1 PMC7680305 32468251

[117] Zamboglou C Carles M Fechter T Kiefer S Reichel K Fassbender TF. Radiomic Features From PSMA PET for non-Invasive Intraprostatic Tumor Discrimination and Characterization in Patients With Intermediate- and High-Risk Prostate Cancer – A Comparison Study With Histology Reference. Theranostics (2019) 9(9):2595–605. doi: 10.7150/thno.32376 PMC6525993 31131055

[118] Li L Shiradkar R Leo P Algohary A Fu P Tirumani SH. A Novel Imaging Based Nomogram for Predicting Post-Surgical Biochemical Recurrence and Adverse Pathology of Prostate Cancer From Pre-Operative Bi-Parametric MRI. EBioMedicine (2021) 63:103163. doi: 10.1016/j.ebiom.2020.103163 33321450 PMC7744939

[119] Reischauer C Patzwahl R Koh D-M Froehlich JM Gutzeit A. Texture Analysis of Apparent Diffusion Coefficient Maps for Treatment Response Assessment in Prostate Cancer Bone Metastases—A Pilot Study. Eur J Radiol (2018) 101:184–90. doi: 10.1016/j.ejrad.2018.02.024 29571795

[120] Musch M Klevecka V Roggenbuck U Kroepfl D. Complications of Pelvic Lymphadenectomy in 1,380 Patients Undergoing Radical Retropubic Prostatectomy Between 1993 and 2006. J Urol (2008) 179(3):923–9. doi: 10.1016/j.juro.2007.10.072 18207170

[121] Fedorov A Schwier M Clunie D Herz C Pieper S Kikinis R. An Annotated Test-Retest Collection of Prostate Multiparametric MRI. Sci Data (2018) 5(1):180281–13. doi: 10.1038/sdata.2018.281 PMC6278692 30512014

[122] Briganti A Larcher A Abdollah F Capitanio U Gallina A Suardi N. Updated Nomogram Predicting Lymph Node Invasion in Patients With Prostate Cancer Undergoing Extended Pelvic Lymph Node Dissection: The Essential Importance of Percentage of Positive Cores. Eur Urol (2011) 61(3):480–7. doi: 10.1016/j.eururo.2011.10.044 22078338

[123] Perk T Chen S Harmon S Lin C Bradshaw T Perlman S. A Statistically Optimized Regional Thresholding Method (SORT) for Bone Lesion Detection in F-18-NaF PET/CT Imaging. Phys Med Biol (2018) 63(22):225018. doi: 10.1088/1361-6560/aaebba 30457117

[124] Ferdinandus J Eppard E Gaertner FC Kürpig S Fimmers R Yordanova A. Predictors of Response to Radioligand Therapy of Metastatic Castrate-Resistant Prostate Cancer With 177Lu-PSMA-617. J Nucl Med (2017) 58(2):312–9. doi: 10.2967/jnumed.116.178228 27587707

[125] Aoki Y Nakayama M Nomura K Tomita Y Nakajima K Yamashina M. The Utility of a Deep Learning-Based Algorithm for Bone Scintigraphy in Patient With Prostate Cancer. Ann Nucl Med (2020) 34(12):926–31. doi: 10.1007/s12149-020-01524-0 32955663

[126] Cheng D-C Liu C-C Hsieh T-C Yen K-Y Kao C-H. Bone Metastasis Detection in the Chest and Pelvis From a Whole-Body Bone Scan Using Deep Learning and a Small Dataset. Electronics (2021) 10(10):1201. doi: 10.3390/electronics10101201

[127] Ntakolia C Diamantis DE Papandrianos N Moustakidis S Papageorgiou EI. A Lightweight Convolutional Neural Network Architecture Applied for Bone Metastasis Classification in Nuclear Medicine: A Case Study on Prostate Cancer Patients. Healthcare (2020) 8(4):493. doi: 10.3390/healthcare8040493 PMC7711827 33217973

[128] Papandrianos N Papageorgiou E Anagnostis A Papageorgiou K. Efficient Bone Metastasis Diagnosis in Bone Scintigraphy Using a Fast Convolutional Neural Network Architecture. Diagnostics (2020) 10(8):532. doi: 10.3390/diagnostics10080532 PMC7459937 32751433

[129] Papandrianos N Papageorgiou E Anagnostis A Papageorgiou K. Bone Metastasis Classification Using Whole Body Images From Prostate Cancer Patients Based on Convolutional Neural Networks Application. PloS One (2020) 15(8):e0237213. doi: 10.1371/journal.pone.0237213 32797099 PMC7428190

[130] Sadik M Hamadeh I Nordblom P Suurkula M Hoglund P Ohlsson M. Computer-Assisted Interpretation of Planar Whole-Body Bone Scans. J Nucl Med (2008) 49(12):1958–65. doi: 10.2967/jnumed.108.055061 18997038

[131] Wuestemann J Hupfeld S Kupitz D Genseke P Schenke S Pech M. Analysis of Bone Scans in Various Tumor Entities Using a Deep-Learning-Based Artificial Neural Network Algorithm—Evaluation of Diagnostic Performance. Cancers (Basel) (2020) 12(9):1–13. doi: 10.3390/cancers12092654 PMC7565494 32957650

[132] Diamantis DE Iakovidis DK Koulaouzidis A. Look-Behind Fully Convolutional Neural Network for Computer-Aided Endoscopy. BioMed Signal Process Control (2019) 49:192–201. doi: 10.1016/j.bspc.2018.12.005

[133] Diamantis DE Koutsiou D-CC Iakovidis DK. Staircase Detection Using a Lightweight Look-Behind Fully Convolutional Neural Network. Cham: Springer International Publishing (2019) p. 522–32.

[134] Bochkovskiy A Wang C-Y Liao H-YM. Yolov4: Optimal Speed and Accuracy of Object Detection. arXiv:200410934 (2020).

[135] Redmon J Divvala S Girshick R Farhadi A. You Only Look Once: Unified, Real-Time Object Detection. IEEE (2016) p:779–88. doi: 10.1109/CVPR.2016.91

[136] Borrelli P Larsson M Ulén J Enqvist O Trägårdh E Poulsen MH. Artificial Intelligence-Based Detection of Lymph Node Metastases by PET/CT Predicts Prostate Cancer-Specific Survival. Clin Physiol Funct Imaging (2021) 41(1):62–7. doi: 10.1111/cpf.12666 32976691

[137] Hartenstein A Lübbe F Baur ADJ Rudolph MM Furth C Brenner W. Prostate Cancer Nodal Staging: Using Deep Learning to Predict 68ga-PSMA-Positivity From CT Imaging Alone. Sci Rep (2020) 10(1):3398. doi: 10.1038/s41598-020-60311-z 32099001 PMC7042227

[138] Masoudi S Mehralivand S Harmon SA Lay N Lindenberg L Mena E. Deep Learning Based Staging of Bone Lesions From Computed Tomography Scans. IEEE Access (2021) 9:87531–42. doi: 10.1109/ACCESS.2021.3074051 PMC8562651 34733603

[139] Lee JJ Yang H Franc BL Iagaru A Davidzon GA. Deep Learning Detection of Prostate Cancer Recurrence With 18F-FACBC (Fluciclovine, Axumin®) Positron Emission Tomography. Eur J Nucl Med Mol Imaging (2020) 47(13):2992–7. doi: 10.1007/s00259-020-04912-w 32556481

[140] Steyerberg EW. Clinical Prediction Models: A Practical Approach to Development, Validation, and Updating. New York, NY: Springer-Verlag (2009).

[141] Johnson WE Li C Rabinovic A. Adjusting Batch Effects in Microarray Expression Data Using Empirical Bayes Methods. Biostatistics (2007) 8(1):118–27. doi: 10.1093/biostatistics/kxj037 16632515

[142] Orlhac F Boughdad S Philippe C Stalla-Bourdillon H Nioche C Champion L. A Postreconstruction Harmonization Method for Multicenter Radiomic Studies in PET. J Nucl Med (2018) 59(8):1321–8. doi: 10.2967/jnumed.117.199935 29301932

[143] Orlhac F Frouin F Nioche C Ayache N Buvat I. Validation of a Method to Compensate Multicenter Effects Affecting CT Radiomics. Radiology (2019) 291(1):53–9. doi: 10.1148/radiol.2019182023 30694160

[144] Mahon RN Ghita M Hugo GD Weiss E. ComBat Harmonization for Radiomic Features in Independent Phantom and Lung Cancer Patient Computed Tomography Datasets. Phys Med Biol (2020) 65(1):015010. doi: 10.1088/1361-6560/ab6177 31835261

[145] Orlhac F Lecler A Savatovski J Goya-Outi J Nioche C Charbonneau F. How Can We Combat Multicenter Variability in MR Radiomics? Validation of a Correction Procedure. Eur Radiol (2021) 31(4):2272–80. doi: 10.1007/s00330-020-07284-9 32975661

[146] Foy JJ Robinson KR Li H Giger ML Al-Hallaq H. Armato rSG. Variation in Algorithm Implementation Across Radiomics Software. J Med Imaging (2018) 5(4):44505. doi: 10.1117/1.JMI.5.4.044505 PMC6278846 30840747

[147] Zwanenburg A Vallières M Abdalah MA Aerts HJWL Anearczyk V Apte A. The Image Biomarker Standardization Initiative: Standardized Quantitative Radiomics for High-Throughput Image-Based Phenotyping. Radiology (2020) 295(2):328–38. doi: 10.1148/radiol.2020191145 PMC7193906 32154773

[148] van Griethuysen JJM Fedorov A Parmar C Hosny A Aucoin N Narayan V. Computational Radiomics System to Decode the Radiographic Phenotype. Cancer Res (2017) 77(21):E104–7. doi: 10.1158/0008-5472.CAN-17-0339 PMC5672828 29092951

[149] Pfaehler E Zwanenburg A de Jong JR Boellaard R. RACAT: An Open Source and Easy to Use Radiomics Calculator Tool. PloS One (2019) 14(2):e0212223. doi: 10.1371/journal.pone.0212223 30785937 PMC6382170

[150] Thomas TS Pachynski RK. Treatment of Advanced Prostate Cancer. Mo Med (2018) 115(2):156–61. PMC6139850 30228709

[151] Ronneberger O Fischer P Brox T. U-Net: Convolutional Networks for Biomedical Image Segmentation. arXiv:150504597 (2015).

[152] Ibtehaz N Rahman MS. MultiResUNet : Rethinking the U-Net Architecture for Multimodal Biomedical Image Segmentation. Neural Netw (2020) 121:74–87. doi: 10.1016/j.neunet.2019.08.025 31536901

[153] Weng Y Zhou T Li Y Qiu X. NAS-Unet: Neural Architecture Search for Medical Image Segmentation. IEEE Access (2019) 7:44247–57. doi: 10.1109/ACCESS.2019.2908991

[154] Kostyszyn D Fechter T Bartl N Grosu AL Gratzke C Sigle A. Convolutional Neural Network Based Deep-Learning Architecture for Intraprostatic Tumour Contouring on PSMA PET Images in Patients With Primary Prostate Cancer. arXiv:200803201 (2020).

[155] Dong X Lei Y Tian S Wang T Patel P Curran WJ. Synthetic MRI-Aided Multi-Organ Segmentation on Male Pelvic CT Using Cycle Consistent Deep Attention Network. Radiother Oncol (2019) 141:192–9. doi: 10.1016/j.radonc.2019.09.028 PMC6899191 31630868

[156] Kazemifar S Balagopal A Nguyen D McGuire S Hannan R Jiang S. Segmentation of the Prostate and Organs at Risk in Male Pelvic CT Images Using Deep Learning. BioMed Phys Eng Express (2018) 4(5):55003. doi: 10.1088/2057-1976/aad100

[157] Liu C Gardner SJ Wen N Elshaikh MA Siddiqui F Movsas B. Automatic Segmentation of the Prostate on CT Images Using Deep Neural Networks (DNN). Int J Radiat Oncol Biol Phys (2019) 104(4):924–32. doi: 10.1016/j.ijrobp.2019.03.017 30890447

[158] Clark T Zhang J Baig S Wong A Haider MA Khalvati F. Fully Automated Segmentation of Prostate Whole Gland and Transition Zone in Diffusion-Weighted MRI Using Convolutional Neural Networks. J Med Imaging (2017) 4(4):41307. doi: 10.1117/1.JMI.4.4.041307 PMC5644511 29057288

[159] Jia H Xia Y Song Y Zhang D Huang H Zhang Y. 3d APA-Net: 3d Adversarial Pyramid Anisotropic Convolutional Network for Prostate Segmentation in MR Images. IEEE Trans Med Imaging (2020) 39(2):447–57. doi: 10.1109/TMI.2019.2928056 31295109

[160] Khan Z Yahya N Alsaih K Ali SSA Meriaudeau F. Evaluation of Deep Neural Networks for Semantic Segmentation of Prostate in T2W MRI. Sensors (2020) 20(11):1–17. doi: 10.3390/s20113183 PMC7309110 32503330

[161] Hinton GE Srivastava N Krizhevsky A Sutskever I Salakhutdinov RR. Improving Neural Networks by Preventing Co-Adaptation of Feature Detectors. arXiv:12070580 (2012).

[162] Ioffe S Szegedy C eds. Batch Normalization: Accelerating Deep Network Training by Reducing Internal Covariate Shift. In: International Conference on Machine Learning. Lille: PMLR.

[163] Castiglioni I Gallivanone F Soda P Avanzo M Stancanello J Aiello M. AI-Based Applications in Hybrid Imaging: How to Build Smart and Truly Multi-Parametric Decision Models for Radiomics. Eur J Nucl Med Mol Imaging (2019) 46(13):2673–99. doi: 10.1007/s00259-019-04414-4 31292700

