# Supplementary material for: Radiomics for Identification and Prediction in Metastatic Prostate Cancer: A Review of Studies
Source: Front Oncol. 2021 Nov 1;11:771787. doi: 10.3389/fonc.2021.771787 (PMC8591174; doi:10.3389/fonc.2021.771787)
Supplement: Supplementary file 1 [file Table_1.docx]

Supplementary Table 1: RQS scores for each item for the reviewed traditional radiomics papers.

|  |  | **RQS Items** | | | | | | | | | | | | | | | |  |  |
| --- | --- | --- | --- | --- | --- | --- | --- | --- | --- | --- | --- | --- | --- | --- | --- | --- | --- | --- | --- |
| **First Author** | **Year** | **1** | **2** | **3** | **4** | **5** | **6** | **7** | **8** | **9** | **10** | **11** | **12** | **13** | **14** | **15** | **16** | **RQS Score** | **RQS (%)** |
| Wang | 2019 | 0 | 0 | 0 | 0 | 3 | 1 | 0 | 1 | 2 | 0 | 0 | 2 | 0 | 0 | 0 | 0 | 9 | 25.00 |
| Acar | 2019 | 0 | 0 | 0 | 0 | -3 | 0 | 0 | 0 | 0 | 0 | 0 | 2 | 0 | 0 | 0 | 0 | -1 | 0 |
| Alongi | 2021 | 0 | 0 | 0 | 0 | 3 | 1 | 0 | 0 | 2 | 0 | 0 | 2 | 2 | 0 | 0 | 0 | 10 | 27.78 |
| Cysouw | 2020 | 0 | 1 | 0 | 0 | 3 | 0 | 0 | 0 | 2 | 0 | 7 | 2 | 2 | 0 | 0 | 0 | 17 | 47.22 |
| Zamboglou | 2019 | 1 | 1 | 1 | 0 | 3 | 0 | 0 | 1 | 1 | 0 | 7 | 2 | 2 | 0 | 0 | 0 | 19 | 52.78 |
| Damascelli | 2021 | 1 | 1 | 0 | 0 | 3 | 0 | 0 | 0 | 0 | 0 | 0 | 2 | 2 | 0 | 0 | 0 | 9 | 25.00 |
| Li | 2021 | 1 | 0 | 0 | 0 | 3 | 1 | 0 | 1 | 2 | 0 | 0 | 5 | 2 | 2 | 0 | 1 | 18 | 50.00 |
| Hou | 2021 | 0 | 0 | 0 | 0 | 3 | 1 | 0 | 1 | 2 | 0 | 0 | 3 | 2 | 0 | 0 | 0 | 12 | 33.33 |
| Zhang | 2020 | 0 | 1 | 0 | 0 | 3 | 1 | 0 | 1 | 1 | 1 | 0 | 2 | 2 | 2 | 0 | 0 | 14 | 38.89 |
| Peeken | 2020 | 1 | 1 | 0 | 0 | 3 | 0 | 0 | 1 | 2 | 2 | 0 | 3 | 2 | 2 | 0 | 0 | 17 | 47.22 |
| Reischauer | 2018 | 1 | 0 | 0 | 0 | -3 | 0 | 0 | 0 | 0 | 0 | 7 | -5 | 0 | 0 | 0 | 0 | 0 | 0.00 |
| Moazemi | 2021 | 0 | 0 | 0 | 0 | 3 | 1 | 0 | 1 | 0 | 0 | 0 | -5 | 0 | 0 | 0 | 0 | 0 | 0.00 |
| Moazemi | 2020 | 0 | 1 | 0 | 0 | -3 | 0 | 0 | 0 | 2 | 0 | 0 | 2 | 0 | 0 | 0 | 0 | 2 | 5.56 |
| Khurshid | 2018 | 1 | 0 | 0 | 0 | 3 | 0 | 0 | 1 | 1 | 0 | 0 | -5 | 2 | 0 | 0 | 0 | 3 | 8.33 |
| Perk | 2018 | 0 | 1 | 0 | 0 | 3 | 1 | 0 | 0 | 2 | 0 | 0 | 2 | 0 | 0 | 0 | 0 | 9 | 25.00 |
| Lin | 2019 | 1 | 0 | 0 | 0 | -3 | 0 | 0 | 0 | 0 | 0 | 7 | -5 | 2 | 0 | 0 | 0 | 2 | 5.56 |
| Hayakawa | 2020 | 0 | 0 | 0 | 0 | -3 | 1 | 0 | 0 | 0 | 0 | 0 | -5 | 0 | 0 | 0 | 0 | -7 | 0 |
